# KCNE4-dependent functional consequences of Kv1.3-related leukocyte physiology

**DOI:** 10.1038/s41598-021-94015-9

**Published:** 2021-07-16

**Authors:** Albert Vallejo-Gracia, Daniel Sastre, Magalí Colomer-Molera, Laura Solé, María Navarro-Pérez, Jesusa Capera, Sara R. Roig, Oriol Pedrós-Gámez, Irene Estadella, Orsolya Szilágyi, Gyorgy Panyi, Péter Hajdú, Antonio Felipe

**Affiliations:** 1 Molecular Physiology Laboratory, Departament de Bioquímica i Biomedicina Molecular, Institut de Biomedicina (IBUB), Universitat de Barcelona, Avda. Diagonal 643, 08028 Barcelona, Spain; 2 Virology and Immunology, Gladstone Institutes, University of California San Francisco, San Francisco, CA 94158 USA; 3 Department of Biomedical Sciences, Colorado State University, Fort Collins, CO 80523 USA; 4 Department of Biophysics and Cell Biology, Faculty of Medicine, University of Debrecen, 400, 1 Egyetem Sq., Debrecen, 4032 Hungary

**Keywords:** Immunology, Neuroscience, Physiology

## Abstract

The voltage-dependent potassium channel Kv1.3 plays essential roles in the immune system, participating in leukocyte activation, proliferation and apoptosis. The regulatory subunit KCNE4 acts as an ancillary peptide of Kv1.3, modulates K^+^ currents and controls channel abundance at the cell surface. KCNE4-dependent regulation of the oligomeric complex fine-tunes the physiological role of Kv1.3. Thus, KCNE4 is crucial for Ca^2+^-dependent Kv1.3-related leukocyte functions. To better understand the role of KCNE4 in the regulation of the immune system, we manipulated its expression in various leukocyte cell lines. Jurkat T lymphocytes exhibit low KCNE4 levels, whereas CY15 dendritic cells, a model of professional antigen-presenting cells, robustly express KCNE4. When the cellular KCNE4 abundance was increased in T cells, the interaction between KCNE4 and Kv1.3 affected important T cell physiological features, such as channel rearrangement in the immunological synapse, cell growth, apoptosis and activation, as indicated by decreased IL-2 production. Conversely, ablation of KCNE4 in dendritic cells augmented proliferation. Furthermore, the LPS-dependent activation of CY15 cells, which induced Kv1.3 but not KCNE4, increased the Kv1.3-KCNE4 ratio and increased the expression of free Kv1.3 without KCNE4 interaction. Our results demonstrate that KCNE4 is a pivotal regulator of the Kv1.3 channelosome, which fine-tunes immune system physiology by modulating Kv1.3-associated leukocyte functions.

## Introduction

Voltage-dependent potassium channels (Kv) control repolarization and resting membrane potential in electrically excitable cells^1^. In addition, Kv are also involved in the proliferation, apoptosis and activation of immune system cells^2,3^. In this scenario, leukocytes express Kv1.3. The specific upregulation of Kv1.3 in effector memory T cells (T_EM_ cells) during immune responses situates the channel as an important physiological mediator^4^. In addition, impaired channel function is concomitant with several autoimmune diseases^4^. Therefore, Kv1.3 is considered a major pharmacological target for immunotherapy. Kv1.3 channels redistribute in the immunological synapse (IS) during T cell receptor (TCR) engagement, thus regulating the Ca^2+^-dependent T cell activation cascade^3,5^. Similarly, Kv1.3 redirects to lipid rafts and caveolar microdomains during cell activation and differentiation^6,7^. Interestingly, these signal transduction platforms form clusters and concentrate at the IS in leukocytes^8^. Thus, lipid raft aggregation occurs during immune activation. The presence of other Kv channels, such as Kv1.1, Kv1.2 and Kv1.6, has also been reported in T cells, but a minor role has been evidenced^9^. However, antigen-presenting cells (APCs), such as macrophages or dendritic cells, express Kv1.5, which tetramerizes with Kv1.3 and modulates specific physiological events^10,11^. Although specific upregulation of Kv1.3 expression is concomitant with dendritic cell maturation and activation, an increased Kv1.5 ratio within the heteromeric complex leads to immunosuppression^11,12^.

Although leukocytes express a limited repertoire of K^+^ channels, their interaction with several regulatory subunits, such as Kvβs or KCNEs, further increases the catalog of functional roles^13,14^. KCNE4 is the fourth member of the KCNE Kv channel regulatory subunit family. This auxiliary subunit, which is present in leukocytes, physically interacts with the Kv1.3 channel, modifying the trafficking and electrophysiological properties of the Kv1.3 channelosome^15–17^. KCNE4 negatively regulates the channel by retaining the complex intracellularly, inhibiting outward K^+^ currents and accelerating inactivation. KCNE4 is modulated during the proliferation and activation of leukocytes, and the stoichiometry of the oligomeric interaction fine-tunes Kv1.3 activity^18,19^.

Given that KCNE4 associates with Kv1.3 in leukocytes undergoing differential regulation and tightly controls channel function in heterologous expression systems^16–19^, we aimed to decipher the role of KCNE4 in immune cell physiology under different scenarios. We investigated two complementary leukocyte models playing crucial roles at the IS during activation. Specifically, we altered the expression of KCNE4 in Jurkat T lymphocytes and CY15 dendritic cells (APCs) and analyzed the functional consequences on Kv1.3-related leukocyte physiology. We found that KCNE4 fine-tunes the immunological response by modulating a number of events, such as delocalization from the IS, IL-2 production in T-cells, APC activation, proliferation and apoptosis. Our data unequivocally situate KCNE4 as a central regulator in leukocytes, indicating that KCNE4 should be considered a promising target in the development of therapeutic strategies for the treatment of Kv1.3-related immunological disorders.

## Results

### Expression of Kv1.3 and KCNE4 in Jurkat T lymphocytes and CY15 dendritic cells

**Figure 1:**
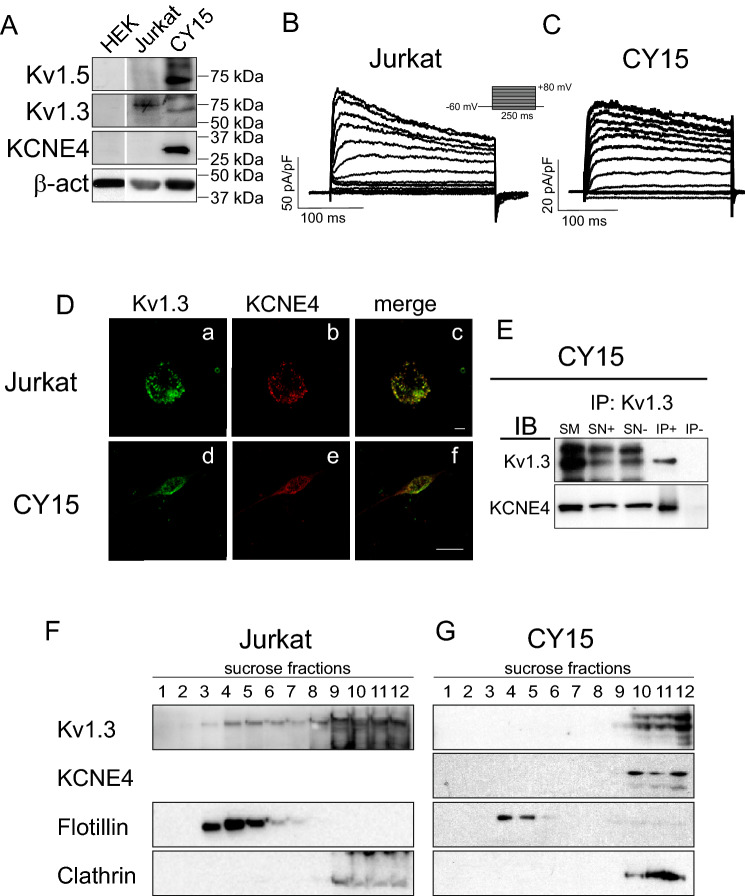
Kv1.3 and KCNE4 are differentially expressed in leukocytes. The presence of Kv1.3 and KCNE4 expression was analyzed in human Jurkat T lymphocytes and mouse CY15 dendritic cells. (**A**) Kv1.3 and KCNE4 protein expression in leukocytes. HEK 293 cells were used as a negative control. Although Jurkat and CY15 dendritic cells shared Kv1.3 and KCNE4 expression, the abundance of KCNE4 in T cells was much lower and minimally detected by western blot. In addition, Kv1.5 was abundantly expressed in CY15 cells. Representative cropped blots, clearly separated by vertical white lines, are shown only for qualitative purposes. Voltage-dependent K^+^ currents were elicited in Jurkat (**B**) and CY15 cells (**C**). Cells were held at -60 mV, and 250 ms pulse potentials were applied as indicated. (**D**) Representative confocal images of Kv1.3 (**Da** and **Dd**, in green) and KCNE4 (**Db** and **De**, in red) in Jurkat T lymphocytes (**Da**–**Dc**) and CY15 dendritic cells (**Dd**–**Df**). Scale bars: 10 µm. Given the limited expression of KCNE4 in T-cells, IPI was performed in Jurkat cells. (**E**) KCNE4 coimmunoprecipitated with Kv1.3 in dendritic cells. Lysates were immunoprecipitated against Kv1.3 (IP: Kv1.3) and immunoblotted (IB) against Kv1.3 and KCNE4. Upper panel: Kv1.3. Lower panel: KCNE4. SM: starting material. SN+: supernatant from the IP+. SN−: supernatant from the IP−. IP+: Immunoprecipitation in the presence of the anti-Kv1.3 antibody. IP−: Immunoprecipitated in the absence of the anti-Kv1.3 antibody. (**F**) Kv1.3 localized in lipid raft fractions from Jurkat T-cells. (**G**) Kv1.3 and KCNE4 did not localize in lipid rafts from CY15 dendritic cells. Lipid rafts were isolated, and low density (1) to high density (12) sucrose gradient fractions were analyzed by western blot. Flotilin indicated low-buoyancy lipid rafts, whereas clathrin identified nonfloating raft fractions.

Although Kv1.3 and KCNE4 associate in HEK 293 cells and are coexpressed in leukocytes, the physiological impact of this interaction in native cells is under debate^15,16,19^. Therefore, we first characterized the presence of Kv1.3 and KCNE4 in leukocytes and determined whether their interaction affects channel behavior in immune cells. As expected, Kv1.3 was present in Jurkat T and CY15 dendritic cells^17^, although KCNE4 expression, as well as Kv1.5 expression, was largely restricted to CY15 dendritic cells (Fig. 1A). In Jurkat T cells, depolarizing pulses elicited classical rapidly outward activating and inactivating Kv1.3 K^+^ current (Fig. 1B), whereas CY15 cells, which are mononuclear phagocytes acting as antigen-presenting cells, exhibited a current with a less inactivating phenotype (Fig. 1C). KCNE4 accelerates Kv1.3 inactivation, but fewer inactivating currents are related to the coexpression of Kv1.5 in dendritic cells (Fig. 1A). To visualize Kv1.3 and KCNE4 localization in immune cells, we performed IPI immunocytochemistry to enhance the KCNE4 signal in T cells. This showed that Kv1.3 and KCNE4 colocalized in CY15 cells and to a lesser extent in Jurkat T cells (Fig. 1D). Coimmunoprecipitation demonstrated that Kv1.3 and KCNE4 were associated with CY15 dendritic cells (Fig. 1E). To determine whether this interaction modifies channel localization, we isolated lipid rafts. Cell activation is known to concentrate Kv1.3 in these microdomains near signaling molecules, and KCNE4 misallocates Kv1.3 from lipid rafts in HEK 293 cells^16,20^. We found that while Kv1.3 targeted lipid rafts in T cells (Fig. 1F), Kv1.3 was not present in floating fractions in CY15 cells (Fig. 1G). Its association to KCNE4, as well as to Kv1.5^20^, would cooperatively cause the absence of Kv1.3 in rafts. Our results demonstrate that leukocytes differentially express Kv1.3 and KCNE4 and that different channelosome compositions influence the biophysical and physiological properties of the complex.

### KCNE4 alters Kv1.3 surface targeting in Jurkat T cells

**Figure 2:**
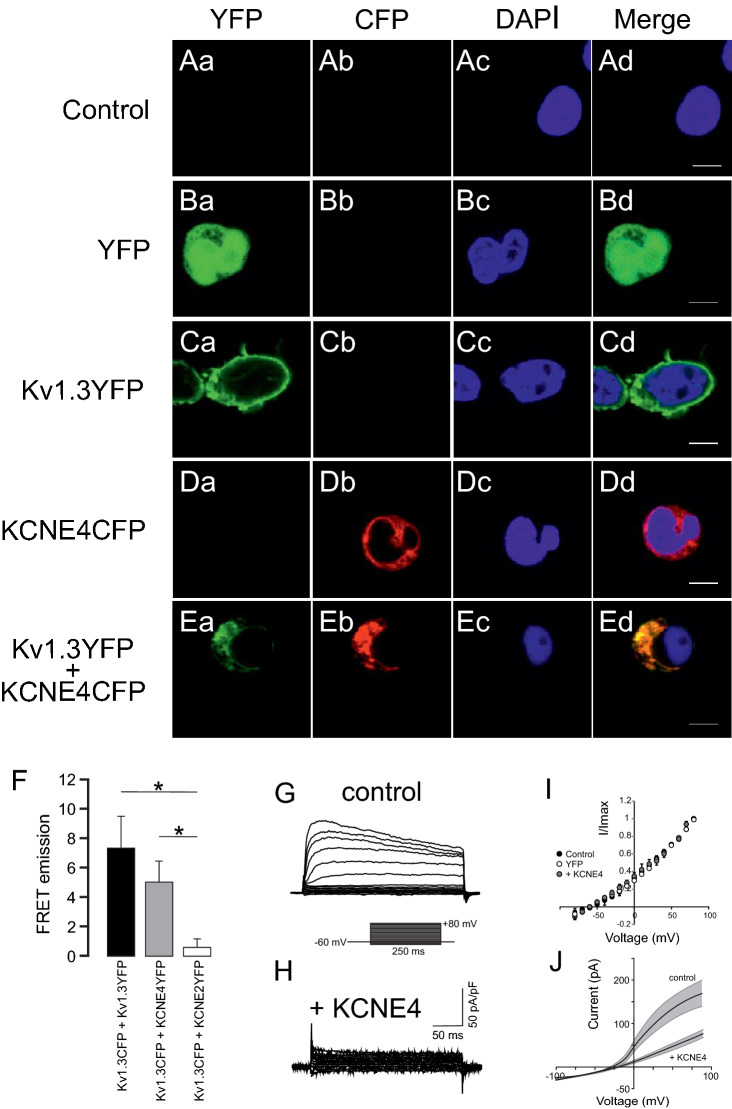
KCNE4 expression impairs Kv1.3 surface expression and inhibits Kv currents in Jurkat T cells. Confocal imaging of Jurkat T cells transfected with YFP, Kv1.3YFP, KCNE4CFP and KCNE4CFP with YFP-Kv1.3. Nuclei were stained with DAPI (blue). (**Aa**–**Ad**) Jurkat nontransfected cells (control). (**Ba**–**Bd**) YFP-transfected cells (YFP). (**Ca**–**Cd**) Kv1.3YFP transfected cells. (**Da**–**Dd**) KCNE4CFP transfected cells. (**Ea**–**Ed**) Kv1.3YFP and KCNE4CFP cotransfected cells. (**Aa**, **Ba**, **Ca**, **Da**, **Ea**) Kv1.3 in green. (**Ab**, **Bb**, **Cb**, **Db**, **Eb**) KCNE4 in red. (**Ac**, **Bc**, **Cc**, **Dc**, **Ec**) DAPI in blue. Merged yellow indicates colocalization between green and red (**Ad**, **Bd**, **Cd**, **Dd**, **Ed**). Scale bar: 5 µm. (**F**) FRET analysis of the Kv1.3-KCNE4 protein interaction by flow cytometry in Jurkat T lymphocytes. Values are mean ± SE, n = 5–7, *p < 0.05 (Student’s *t*-test). Kv1.3CFP-Kv1.3YFP was used as a positive control, whereas Kv1.3CFP-KCNE2YFP served as the negative control. (**G**, **H**) Representative current traces obtained from control Jurkat T cells (**G**) and cells positively transfected with KCNE4CFP (**H**). Cells were clamped at -60 mV, and K^+^ currents elicited by 250 ms voltage steps from -80 mV to +80 mV in 10-mV increments. (**I**) I/Imax plotted against voltage (mV). Black circles, control cells (n = 21); white circles, YFP (n = 4); gray circles, + KCNE4CFP cells (n = 6). (**J**) Kv currents recorded in the whole-cell configuration by depolarizing ramps from -100 to +100 mV. Each black trace represents the average ± SE (shadowed in gray) of several ramps. Traces shown for control Jurkat T cells (n = 22) and + KCNE4CFP (n = 5).

KCNE4 exhibited limited expression in Jurkat cells but its mRNA is tightly regulated in these lymphocytes^18^. To understand how KCNE4 upregulation alters T cell physiology, Jurkat T cells (Control) were electroporated with YFP or Kv1.3YFP in the absence or presence of KCNE4CFP (Kv1.3YFP + KCNE4CFP) (Fig. 2). In the absence of KCNE4, while Kv1.3YFP appeared primarily at the surface of cells (Fig. 2Ca–Cd), endogenous Kv1.3 exhibited a more uniform distribution (Supplementary Fig. 1). KCNE4CFP was mostly intracellular, as expected (Fig. 2Da–Dd). Similar to that observed in CY15 dendritic cells (Fig. 1D), the presence of KCNE4 triggered intracellular retention of Kv1.3 (Fig. 2Ea–Ed), and FACS-based FRET demonstrated molecular proximity between Kv1.3YFP and KCNE4CFP in Jurkat cells (Fig. 2F and Supplemental Fig. 2). In addition, the presence of KCNE4CFP inhibited K^+^ currents in T lymphocytes (Fig. 2G,H). KCNE4 triggered a notable decrease in Kv1.3 currents with minor effects on steady-state activation (Fig. 2I,J).

### Increased KCNE4 profoundly affects Jurkat T lymphocyte physiology

**Figure 3:**
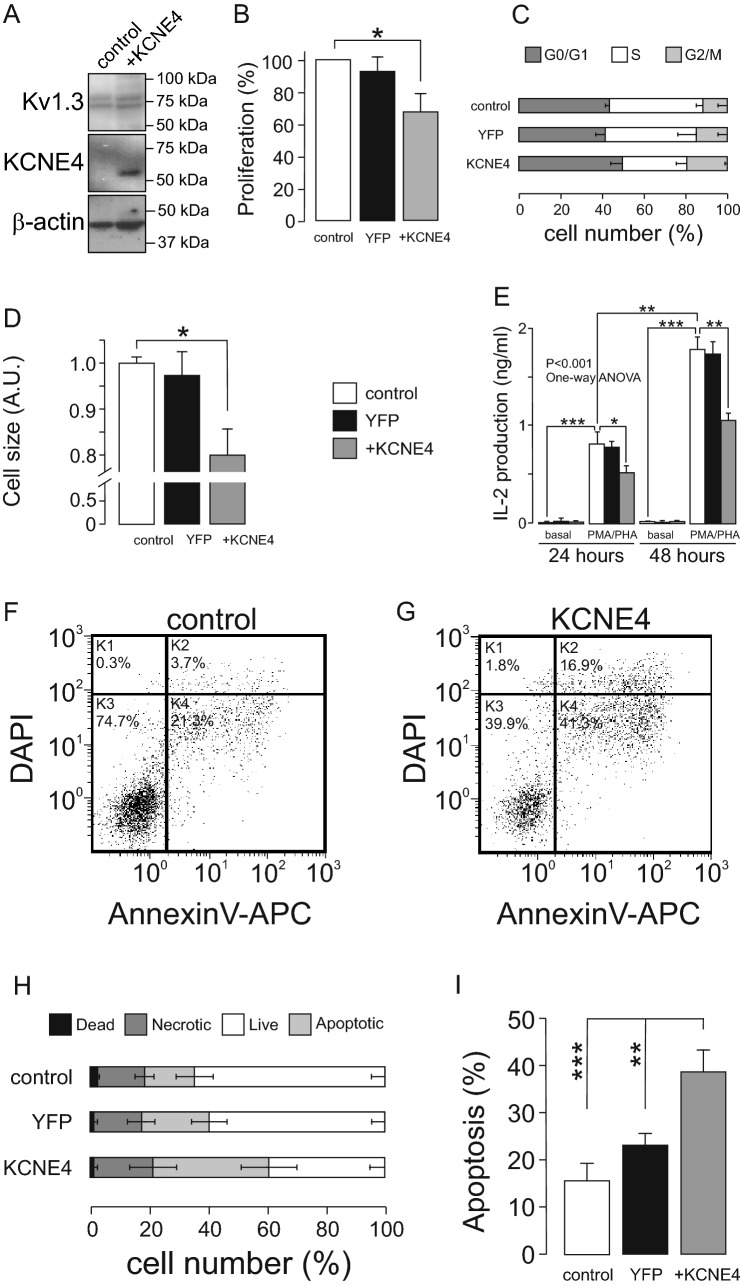
KCNE4 overexpression modulates Kv1.3-related physiological events in Jurkat T lymphocytes. Jurkat T cells were electroporated with KCNE4CFP, and positively transfected cells were selected for specific assays. (**A**) Kv1.3 and KCNE4 expression in Jurkat cells. (**B**) Percentage of Jurkat T cell proliferation. Cells were serum starved overnight and cultured for an additional 24 h in the presence of FBS. The alamarBlue dye was used. *p < 0.05, vs. control; n = 5–7 independent experiments (Student’s *t*-test). (**C**) Cell cycle analysis with Hoechst 33342 Jurkat T cells. Cell cycle distribution (% of cells in each phase) after 24 h in the presence of FBS. G_0_/G_1_-phase, dark gray bars; S-phase, white bars; G_2_/M-phase of the cell cycle, light gray bars. Mean ± SE (n = 3 independent experiments). (**D**) Relative cell size of Jurkat cells measured in a Countess™ automated cell counter. The size of control cells corresponded to a diameter of 10.9 ± 0.6 µm. Values are the mean ± SE from n = 11 independent experiments (*p < 0.05, + KCNE4 vs. control; Student’s *t*-test). (**E**) KCNE4CFP overexpression hampered IL-2 production in activated Jurkat T lymphocytes. Cells were cultured in the presence or absence of PMA (80 nM) and PHA (5 mg/ml) for 24 and 48 h. IL-2 production (ng/ml) from basal and PMA/PHA-stimulated cells was measured. Values are mean ± SE of n = 5–8 different experiments. *p < 0.05; **p < 0.01; ***p < 0.001 (one-way ANOVA and Tukey’s post hoc test). (**F**–**I**) The presence of KCNE4CFP increased apoptosis in Jurkat T cells. (**F**) Representative results of the apoptosis assay (Annexin V-APC—DAPI assay) in control Jurkat cells. (**G**) Apoptosis experiments in KCNE4CFP-positive Jurkat cells. (**H**) Percentage of control Jurkat T cells transfected with YFP and KCNE4CFP under different conditions. (**I**) Percentage of apoptotic control, YFP and KCNE4CFP cells. Values are the mean ± SE from n = 4–6 independent experiments; **p < 0.01; ***p < 0.001, Student’s *t*-test). Panels (**B**, **D**, **E** and **G**) White bars, control Jurkat T-cells; black bars, YFP-transfected cells; gray bars, KCNE4CFP-positive cells.

Increasing KCNE4 levels in T cells decreased Kv1.3 currents with a concomitant increase in intracellular retention of the channel. Therefore, we monitored the effect of KCNE4 overexpression on Kv1.3-related physiological events. Although the KCNE4 overexpression did not remodeled endogenous Kv1.3 expression (Fig. 3A), the proliferation of Jurkat cells decreased by approximately 40% with cells retained at the G_0_/G_1_ phase of the cell cycle (Fig. 3B,C and Supplementary Fig. 3). In addition, KCNE4CFP-positive Jurkat cells exhibited a smaller size, as observed by the analysis of cell capacitance (control Jurkat: 6.1 ± 0.6 pF, n = 19; KCNE4: 2.8 ± 0.9 pF, n = 9, p < 0.01, Student’s t-test) and using a Countess™ automatic cell counter (Fig. 3D). Because low proliferative rates and small cell sizes characterize unresponsive T cells^21^, we analyzed activation under inflammatory conditions. Cell activation, which was measured by IL-2 production in response to PMA/PHA stimulation, was reduced in KCNE4CFP-transfected Jurkat cells (Fig. 3E). Evidence demonstrates that KCNE4 notably reduces, but does not completely annihilate, the insertion of functional Kv1.3 channels into the cell surface^16,19^. Therefore, the presence of 10 nM Margatoxin, a Kv1.3 inhibitor, synergistically affected the KCNE4-dependent reduction of cell activation (Supplementary Fig. 4). Finally, the number of cells undergoing apoptosis—early or late—was augmented twofold by KCNE4 expression (Fig. 3F–I). Taken together, these data demonstrated that specifically increasing the abundance of KCNE4 hampered proliferation and activation and increased T cell apoptosis.

### KCNE4 impairs the Kv1.3 rearrangement in the immunological synapse

**Figure 4:**
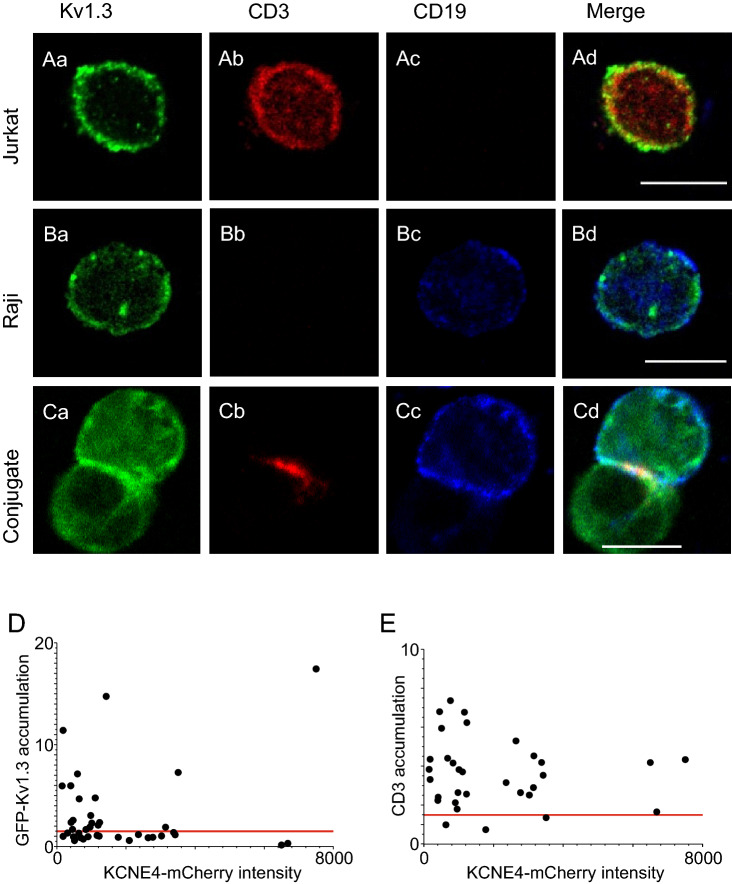
KCNE4 impaired Kv1.3 accumulation in the IS but did not disrupt IS formation. Human Jurkat T lymphocytes and human Raji B lymphocytes were used to generate cell conjugates. (**A**–**C**) Activated B-cells (10 µg/mL SEE toxin) were cocultured in the absence (**Ba**–**Bd**) or presence (**Ca**–**Cd**) of Jurkat cells, and confocal images were obtained. (**Aa**–**Ad**) Jurkat T-cells in the absence of B cells. Endogenous Kv1.3 (green), CD3 (marker of T-cells, red), and CD19 (marker of B-cells, blue) were detected. (**Ad**, **Bd**, **Cd**) merge panels. Note that triple colocalization (white) in Cd localizes Kv1.3 in the IS, as identified by CD3 staining (**Cb**). Bars are 20 µm. (**D**) Accumulation ratio of mGFP-Kv1.3 at the IS vs. KCNE4-mCherry total intensity (n = 40). (**E**) CD3 recruitment into the IS vs. KCNE4 intensity. The horizontal red line represents the threshold level (1.5) for Kv1.3 and CD3 accumulation in the IS. Values greater or less than 1.5 indicated positive or negative accumulation of proteins at the IS, respectively.

The interaction of professional antigen-presenting cells (APCs) with T cells creates an immunological synapse (IS) that leads to T lymphocyte activation^22^. Kv1.3 and CD3 molecules are located in overlapping membrane domains in Jurkat T cells and redistribute into the IS upon activation^5,23,24^. The IS concentrates the lipid raft membrane microdomains to which Kv1.3 is targeted, and KCNE4 impairs channel localization in rafts^8,16^. Therefore, we asked whether increased KCNE4 expression in T cells would alter Kv1.3 targeting to lipid rafts during IS formation between Jurkat cells and APCs. In conjugates between human Jurkat T cells and activated human Raji B cells, the T cell Kv1.3 channels are translocated into the IS, highlighted by CD3 staining (Fig. 4A–C). However, Kv1.3 accumulation at the IS was dependent on the abundance of KCNE4. In the absence of KCNE4-mCherry, 46% of T cells showed Kv1.3 polarization (Fig. 4D). In accordance with previous lipid-raft results^16,25^, an increase in KCNE4 expression reduced Kv1.3 recruitment into the IS (Fig. 4D). CD3 accumulation at the IS occurred in 91.6% of the cells and was largely unaffected by KCNE4 (Fig. 4E). This finding indicates that although IS formation was unaffected by the expression of KCNE4, the spatial location of Kv1.3 at IS was impaired by the presence of KCNE4.

### KCNE4 downregulation in CY15 dendritic cells

**Figure 5:**
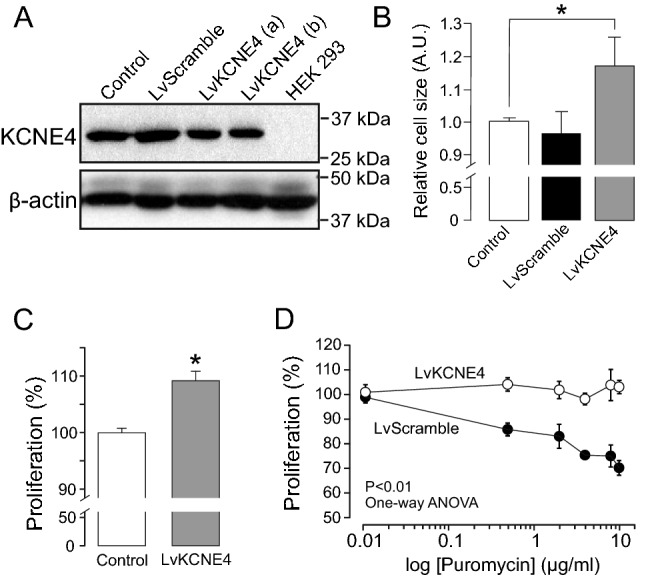
KCNE4 depletion alters CY15 dendritic cell size and proliferation. CY15 cells were treated with shRNA lentiviral particles against KCNE4, and FBS-dependent proliferation was analyzed. (**A**) Representative western blot of KCNE4 protein expression in control CY15 dendritic cells treated with LvKCNE4 and LvScramble particles. HEK 293 cells were used as a negative control. β-actin was used as a loading control. Results from two independent clones (a and b) are shown. (**B**) Relative cell size of control CY15 dendritic cells (white bars), LvScramble (black bars) and LvKCNE4 (gray bars) cells. Control size was 10.4 ± 0.3 µm diameter. *p < 0.05 vs control; n = 5–8 independent experiments (Student’s *t*-test). (C) Percentage of FBS-dependent proliferation during 24 h of control CY15 (white bar) and LvKCNE4 (gray bar) dendritic cells in the absence of puromycin (*p < 0.05, vs control; n = 5 independent experiments; Student’s *t*-test). (D) FBS-dependent proliferation of LvScramble (white circle) and LvKCNE4 (gray circle) dendritic cells over 24 h in the presence of increasing puromycin concentrations. (p < 0.01 one-way ANOVA, n = 5–8). Values are the mean ± SE.

Our data demonstrated that specific increases in KCNE4 negatively altered Kv1.3-related events in T lymphocyte physiology. APCs play essential roles by interacting with T cells during the immune response^22^. Unlike T cells, APCs, such as dendritic cells and macrophages, express notable levels of KCNE4^16,17^. Therefore, we next wanted to monitor physiological alterations in Kv1.3-related events when KCNE4 was specifically suppressed. Thus, we generated a KCNE4 knockdown cell line of CY15 dendritic cells by lentiviral infection (LvKCNE4). Although the efficacy of KCNE4 suppression was only partial (approximately 25%; Fig. 5A), knockdown significantly increased cell size as evaluated using a Countess™ automated cell counter (Fig. 5B). In addition, LvKCNE4 cells exhibited slightly, but significantly (p > 0.05 vs. control), higher proliferation rates than control and LvScramble cells (Fig. 5C). Notably, these proliferation assays were performed in the absence of puromycin because control cells contained no puromycin-resistant lentiviral particles. However, in alternative assays, steadily increasing puromycin concentrations decreased the rate of proliferation of LvScramble cells more than LvKCNE4 cells (P < 0.01, one-way ANOVA) (Fig. 5D).

**Figure 6:**
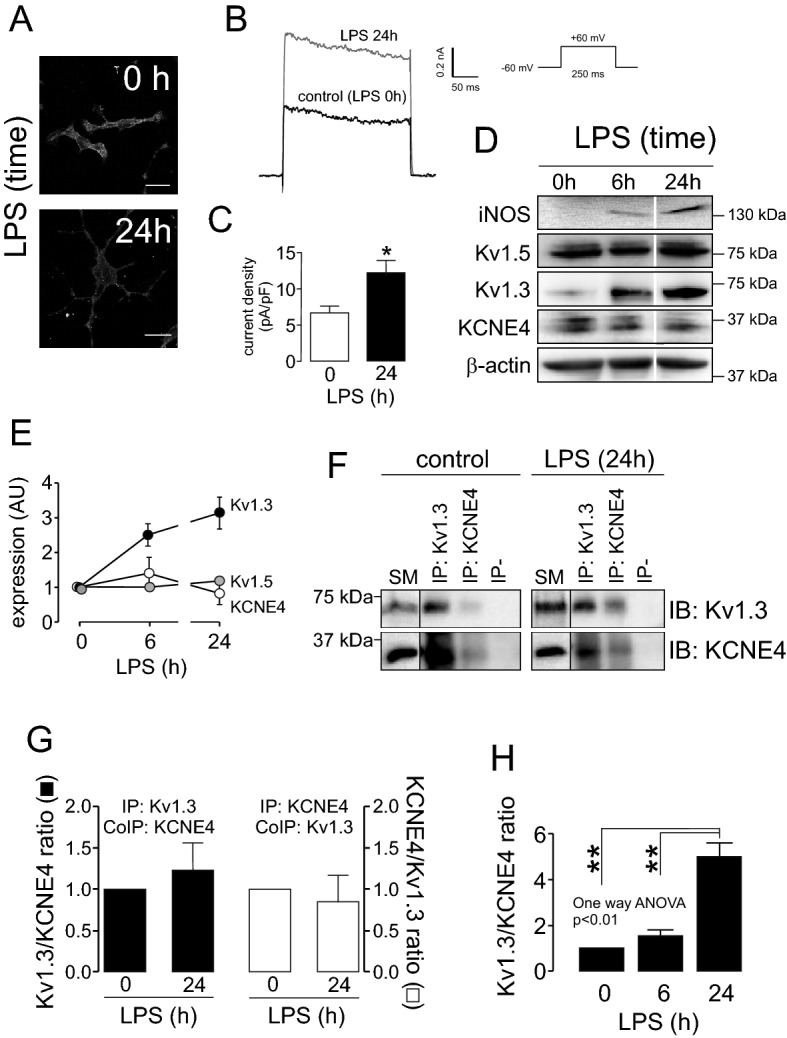
LPS-dependent activation increases the Kv1.3/KCNE4 ratio in CY15 dendritic cells. Cells were treated for 24 h with LPS (100 ng/ml), and the protein expression of selected K^+^ channel proteins was studied at 0, 6 and 24 h. (**A**) Confocal images of CY15 cells stained with Kv1.3 upon LPS treatment. Scale bars: 20 µm. (**B**) Representative voltage-dependent K+ currents elicited in CY15 cells treated with (LPS 24 h) or without (LPS 0 h) LPS during 24 h. Cells were held at -60 mV and 250 ms pulses to +60 mV were applied. (**C**) Peak current densitiy (pA/pF) of K^+^ currents from CY15 cells in the absence (0 h) or the presence (24 h) of LPS. White bars, LPS 0 h (control); black bars, LPS 24 h. Values are the mean ± SE of 4–6 cells. *p < 0.05 vs 0 h, Student’s t test. (**D**) Representative western blot of Kv1.5, Kv1.3 and KCNE4. The expression of iNOS monitored cellular activation in the presence of LPS. β-actin was used as a loading control. Representative cropped blots, clearly delineated by vertical white lines, are shown. (**E**) Relative expression of protein abundance. Kv1.3, black circles; Kv1.5, gray circles; KCNE4, white circles. (**F**) Coimmunoprecipitation of Kv1.3 and KCNE4 in CY15 cells in the absence (control) or presence of LPS for 24 h. Lysates were immunoprecipitated against Kv1.3 (IP: Kv1.3) and KCNE4 (IP: KCNE4) and immunoblotted (IB) against Kv1.3 and KCNE4. Upper panel: Kv1.3. Lower panel: KCNE4. SM: starting material. IP−: Immunoprecipitated in absence of antibodies. Representative cropped blots from SM, clearly delineated by vertical black lines, are shown. (G) Relative coimmunoprecipitation of KCNE4 with Kv1.3 (Kv1.3/KCNE4 ratio) and Kv1.3 with KCNE4 (KCNE4/Kv1.3 ratio). Black bars, Kv1.3 was immunoprecipitated (IP: Kv1.3), and the associated KCNE4 (CoIP: KCNE4) was analyzed. White bars, KCNE4 was immunoprecipitated (IP: KCNE4), and the association of Kv1.3 (CoIP: Kv1.3) was analyzed. (H) The Kv1.3/KCNE4 ratio calculated from the protein expression of Kv1.3 and KCNE4 in LPS-treated CY15 dendritic cells. Values are mean ± SE of n = 4 independent samples. **p < 0.01 One-way ANOVA with post hoc Tukey’s test.

**Figure 7:**
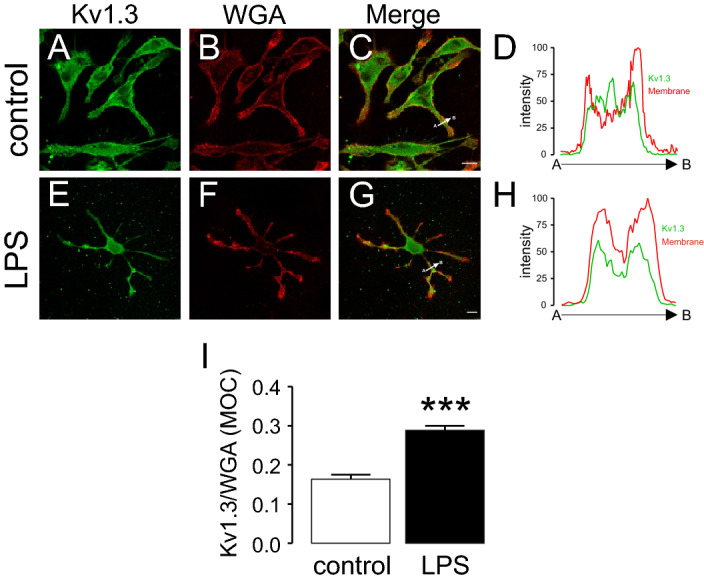
LPS-dependent activation of CY15 dendritic cells increases the abundance of Kv1.3 at the cell surface. CY15 cells were incubated in the presence (LPS) or the absence (control) of LPS for 24 h. Cells were first stained with WGA (membrane marker) and then immunolabeled against Kv1.3. (**A**–**D**) Control cells in the absence of LPS. (**E**–**H**) Cells treated with LPS. Green, Kv1.3; red, WGA; merged panels show colocalization between green and red. (**D**, **H**) Histogram of the pixel by pixel analysis of the section indicated by the arrow in (**C**, **G**), respectively. Bars represent 10 μm. (**I**) Mander's overlap coefficient (MOC) quantifying the degree of colocalization between Kv1.3 and membrane surface (WGA) staining. White bar, control; black bar, LPS. Values are mean ± SE of n > 30 cells. ***p < 0.01 vs control (Student’s t-test).

Finally, we analyzed whether physiological activation of CY15 cells triggered changes in the ratio of Kv1.3/KCNE4 interaction (Fig. 6). CY15 dendritic cells were incubated with 100 ng/ml LPS, and activation was monitored by changes in cell morphology. LPS-dependent activated cells (24 h) exhibited a more stellate shape than control (0 h) cells (Fig. 6A). Furthermore, LPS increased voltage-dependent K^+^ currents of CY15 cells (Fig. 6B,C). Activation was further verified by iNOS expression (Fig. 6D). Under this scenario, several channel proteins, such as Kv1.3, Kv1.5 and KCNE4, were also analyzed (Fig. 6D). Upon LPS treatment, the expression of Kv1.3 abundantly increased, whereas KCNE4 and Kv1.5 expression was unchanged (Fig. 6E). To investigate further, we performed coimmunoprecipitation between Kv1.3 and KCNE4 upon LPS-dependent activation. Our data confirmed that although Kv1.3 increased, the number of channels interacting with KCNE4 was similar during activation (Fig. 6F,G). Thus, the Kv1.3/KCNE4 ratio upon LPS-dependent activation was notably augmented approximately fivefold (Fig. 6H). Our data indicated that CY15 cell activation is concomitant with elevated amounts of Kv1.3 free of interaction with KCNE4. KCNE4 efficiently retains Kv1.3 intracellular. Thus, we wondered whether KCNE4-free Kv1.3 would target the cell surface. In this scenario, LPS-dependent CY15 cell activation concomitantly increased the amount of Kv1.3 colocalized with the membrane marker (Fig. 7).

## Discussion

Our work shows that KCNE4 controls the membrane abundance and the spatial localization of Kv1.3 channels in immune cells, such as T lymphocytes and dendritic cells. We have demonstrated for the first time that KCNE4 association with Kv1.3 fine-tunes channel-related physiological events in leukocytes. The present study is the first to address how changes in the composition of the channelosome affect the physiological responsiveness of immune cells, demonstrating that proliferation, activation, apoptosis and cell size, as well as the spatial rearrangement of the channel at IS, are modulated by KCNE4. Our data pave the way for a better understanding of immune system physiology aiming to develop therapeutic approaches without compromising the protective immune response mediated by other lymphoid subsets^4^.

Formation of the IS is a crucial step in T cell activation^8^. The Kv1.3 channel accumulates at the IS formed between a T and an APC^5,23,24^. We found that the presence of the auxiliary KCNE4 subunit impaired Kv1.3 accumulation in the IS without apparent effects on IS formation. Evidence suggests that in systemic lupus erythematosus (SLE), defective temporal and spatial Kv1.3 distribution is associated with abnormal T cell function^23^. Therefore, it would be interesting to analyze the contribution of KCNE4 to SLE and other autoimmune diseases. Similarly, other proteins, such as PSD-95 and SAP97, which bind to the C-terminus of Kv1.3 via a PDZ-binding motif, regulate the polarized cell surface expression and localization of Kv channels in Jurkat T cells. PSD-95 participates in Kv1.3 rearrangement into the IS, indicating its important role in human T cell activation^25^. PSD-95 recruits Kv1.3 into lipid raft microdomains, protecting the channel against endocytosis^26^. In this context, Kv1.3 and KCNE4 also form functional complexes via both C-terminal domains^15,17^. In addition, the KCNE4 C-terminal domain situates CaM, which plays roles during the immune response, as a major partner in this scenario^15^. Evidence therefore suggests that KCNE4, via interactions and competitions, with the C-terminal domain of Kv1.3 would balance the final fate of the channel.

In addition to modulating spatial reorganization, the KCNE4 association clearly affects the Kv1.3-related physiological state. IL-2 plays a critical role in T cell proliferation, activation, and differentiation. The K^+^ channel blocker charybdotoxin decreases IL-2 production and proliferation in human T cells^27^, similarly to what we found incubating cells MgTx. Our data also demonstrated that by augmenting the expression of KCNE4, IL-2 secretion is impaired in PMA/PHA-stimulated T lymphocytes concomitantly with an inhibition of cell growth. Furthermore, the KCNE4-dependent IL-2 reduction was synergistically affected by inhibiting Kv1.3 cell surface channels with MgTx. Interestingly, the IL-2 receptor in activated T lymphocytes has been targeted in several clinical trials for rheumatoid arthritis or to prevent rejection in transplants^28^. Our results indicate that KCNE4 could also be considered a target for immunosuppression to control T cell activation.

We also found that KCNE4 overexpression induced apoptosis and reduced T-cell size. This fact is consistent with a hallmark of cancer, which links apoptosis with the loss of cell volume or cell shrinkage^29^. Kv1.3 plays a dual function in cell survival^30^. Although the plasma membrane-localized channel has been connected with proliferation, mitochondrial Kv1.3 (mitoKv1.3) is associated with apoptosis through interaction with the pro-apoptotic protein Bax^31^. It is tempting to speculate that KCNE4 controls the localization and action of the surface channel without altering the mitochondrial channel. If this were the case, the oscillations in K^+^ and, consequently, differential Ca^2+^ triggers would determine whether Kv1.3 promotes apoptosis or cell growth^29,32^, and KCNE4 would determine the balance between these two entities.

Our work further investigated the consequences of KCNE4 suppression in dendritic cells. Immature DCs are extremely effective in capturing antigens, whereas mature DCs are very efficient in presenting antigens^33^. Unlike Kv1.5, Kv1.3 expression increases during DC maturation and is the predominant outward K^+^ current in mature dendritic cells^11^. Moreover, Kv1.3 and Kv1.3/Kv1.5 heteromeric channels modulate specific phagocyte responses^34,35^. Interestingly, the fact that KCNE4 repression triggered an increase in both cell size and proliferation rate in APC cells agrees with the opposite phenomenon observed in T cells. The function of KCNE4 in Kv1.3-related function is such that upon inflammatory insults in CY15 cells, the Kv1.3/KCNE4 ratio increased, and the relative levels of Kv1.3 interacting with KCNE4 decreased. Therefore, upon activation, more Kv1.3 should be freely active at the cell surface, contributing to the inflammatory response. Concomitantly, Kv1.3/Kv1.5 heterotetramers contain more Kv1.3 subunits in activated mononuclear phagocytes. This complex scenario would explain why effective Kv1.3 blockade hampers LPS-dependent activation in macrophages^34^. However, because KCNE4 only associates to Kv1.3, the effects of this regulatory peptide on hybrid channels deserves further research. Interestingly, all this experimental evidence is concomitant with cell volume changes that characterize the responsiveness of leukocytes^21^.

In summary, our study demonstrated that KCNE4 plays a pivotal role in regulating Kv1.3 channel expression and function in leukocytes. By remodeling KCNE4 abundance, immune cells modify the Kv1.3/KCNE4 ratio in the Kv1.3 channelosome and fine-tune several physiological features during the inflammatory response. Our results situate the regulatory subunit KCNE4 as a target for therapeutic approaches at the onset of autoimmune diseases, which are characterized by aberrant activity of Kv1.3 in leukocytes.

## Methods

### Expression plasmids

rKv1.3 in pRcCMV was provided by Todd C. Holmes (University of California, Irvine, CA). mKCNE4 in pSGEM was from Michael Sanguinetti (University of Utah, Salt Lake City, UT). hKCNE2 in pHA was obtained from Susana de la Luna (CRG, Barcelona, Spain). Kv1.3, KCNE4 and KCNE2 were subcloned into pEYFP-C1 and pECFP-C1 (Kv1.3, Clontech) as well as pEYFP-N1 and pECFP-N1 (KCNE4 and KCNE2, Clontech). Constructs were verified by sequencing and previously characterized^15–17^. The membrane-localized YFP-CFP tandem construct was kindly provided by F. Barros and P. de la Peña (Universidad de Oviedo, Oviedo, Spain). piRES-EGFP-hKCNE4-HA was obtained from Alfred L. George (Northwestern University, Chicago, IL). Immunological studies were performed with retroviral vector plasmids containing mGFP-Kv1.3 or cloned hKCNE4 in pmCherry-C1 (Addgene).

### Cell culture, transient transfection and lentiviral infection

Jurkat T lymphocytes and the murine CY15 dendritic cell line (kindly provided by Dr. R. Vicente, Universitat Pompeu Fabra, Spain) were cultured in RPMI culture medium (Lonza) containing 10% heat-inactivated FBS and supplemented with 10,000 U/ml penicillin, 100 µg/ml streptomycin and 2 mM L-glutamine (Gibco)^17^. Jurkat T cells were grown in 25 or 75 cm^2^ flasks, and CY15 dendritic cells were grown in 150-mm culture dishes. Treatments were performed for 24 h in culture media. Jurkat T lymphocytes were stimulated with a combination of 5 mg/ml phytohematoxylin (PHA, Sigma–Aldrich), 80 nM phorbol ester (PMA, Sigma–Aldrich)^36^. Controls with DMSO, which is used as a vehicle for PMA, were used to eliminate possible nonspecific side effects. In some experiments, CY15 dendritic cells were incubated with 100 ng/ml lipopolysaccharide (LPS, Sigma–Aldrich) for 24 h.

Jurkat T lymphocytes were transiently transfected by using the Gene Pulser® II Electroporation System (Bio-Rad). Briefly, 1 × 10^6^ cells per electroporation were resuspended in a 0.4-cm cuvette in 800 µl RPMI medium containing 40 µg of plasmid DNA. Microporator parameters were set to pulse voltages of 350 mV and 975 µF. Electroporated cells were cultivated in 10 ml RPMI with standard supplements. After 24 h, positively transfected cells were sorted by using the 488 nm laser of FACSAria FUSION (BD Bioscience) equipment. In some experiments, cells were incubated with 10 nM Margatoxin (MgTx, Alomone) for the IL-2 production assay.

To knock down KCNE4 in CY15 dendritic cells, KCNE4 shRNA (mouse) lentiviral (LTV) particles were used (Santa Cruz Biotechnology). Cells (1 × 10^6^) were cultured in 10 cm plates and infected with 50,000 infectious units in the presence of polybrene (2 µg/ml). Twenty-four hours after transfection, this medium was replaced with regular medium supplemented with 10 µg/ml puromycin (Sigma) for clone selection. Selected individual clones were maintained with 2 µg/ml puromycin^12^. Specific KCNE4 silencing was confirmed by protein expression (LvKCNE4). Control shRNA LTV particles containing a scramble sequence were also used (LvScramble).

### Protein extraction, coimmunoprecipitation and western blotting

Cells were washed twice in cold PBS and lysed on ice with lysis solution (1% Triton X-100, 10% glycerol, 50 mM HEPES pH 7.2, 150 mM NaCl) supplemented with 1 µg/ml aprotinin, 1 µg/ml leupeptin, 1 µg/ml pepstatin and 1 mM phenylmethylsulfonyl fluoride as protease inhibitors. Homogenates were centrifuged at 3000×*g* for 10 min, and the supernatant was collected. Protein content was determined using the Bio-Rad Protein Assay (Bio-Rad)^15,17^. For coimmunoprecipitation, 2 mg of protein was brought up to 500 µl with lysis buffer for immunoprecipitation (NaCl 150 mM, HEPES 50 mM, Triton X-100 1%, pH 7.4) supplemented with protease inhibitors. Samples were precleared with 50 µl of protein G-Sepharose beads for 2 h at 4 °C with gentle mixing. The beads were then removed by centrifugation at 1000×*g* for 30 s at 4 °C. The sample was then incubated for 2 h with 4 µg/mg protein of the desired antibody at room temperature (RT) with gentle mixing. Fifty microliters of protein-G-Sepharose was added to each sample for 2 h at 4 °C. The beads were removed by centrifugation at 1000×*g* for 30 s at 4 °C, washed four times in PBS, and resuspended in 100 µl of SDS sample buffer^15,17^. Protein samples (50 µg) and immunoprecipitates were boiled in Laemmli SDS loading buffer and separated by 10% SDS-PAGE. Next, samples were transferred to nitrocellulose membranes (Immobilon-P; Millipore) and blocked in 0.05% Tween-20-PBS supplemented with 5% dry milk before immunoreaction. Filters were immunoblotted with antibodies against Kv1.3 (1/200, Neuromab), Kv1.5 (1/500, Alomone), KCNE4 (1/500, BD Transduction) and iNOS (1/200, Santa Cruz Biotechnology). Anti-flotillin antibody was used as a marker of lipid raft fractions (1/1000, BD Transduction), and anti-clathrin antibody was used to characterize nonfloating fractions (1/1000, Chemicon). Anti-β-actin antibody was used as a loading control (1/50,000, Sigma). Secondary antibodies were obtained from Bio-Rad (anti-rabbit, 1/3000; anti-mouse, 1/10,000). Densitometric analysis was performed using ImageJ software (v1.53.e, NIH, USA. https://imagej.nih.gov/ij/).

### Immunocytochemistry

For Jurkat T cell immunocytochemistry experiments, 2.5–3.5 × 10^6^ cells/well were seeded in a 12-multiwell dish (Cultek) in poly-l-lysine (Sigma)-coated coverslips with starvation media (RPMI medium with 2 mM l-glutamine and 25 mM HEPES). Twenty-four hours after seeding, immunocytochemistry was performed. Cells were washed twice with PBS without K^+^ (PBS-K^+^) and fixed with 4% paraformaldehyde (PFA) (Sigma) in PBS-K^+^ for 10 min at RT. Next, the cells were washed three times with PBS-K^+^ for 5 min and permeabilized for 20 min with 0.1% Triton X-100 PBS-K^+^. After three washes with 0.05% Triton X-100 PBS-K^+^, the cells were further incubated with the blocking solution (5% milk, 10% goat serum in 0.05% Triton X-100 PBS-K^+^) for 1 h at RT. Next, the cells were incubated with anti-KCNE4 (1/50, Proteintech) or anti-Kv1.3 extracellular (1/150, Alomone) antibodies with 10% goat serum in 0.05% Triton X-100 PBS-K^+^ for 1 h 45 min at RT. Then, cells were washed three times with 0.05% Triton X-100 PBS-K^+^ and then incubated with the secondary antibody (1/150 Cya5 or Alexa-568, Molecular Probes) with 1% BSA 0.05% Triton X-100 PBS-K^+^ for 45 min at RT. Finally, the cells were washed three times with PBS-K^+^ for 5 min, and the coverslips were mounted on microscope slides (Acefesa) with a drop of mounting media (Molecular Probes). Coverslips were dried at RT for at least one day before being observed by microscopy^17^. In the case of transfected cells, 24 h after transfection, cells were washed in PBS-K^+^ and fixed with 4% PFA in PBS-K^+^ for 10 min. After three washes with PBS-K^+^ for 5 min, cells were incubated for 3 min with DAPI (1 µg/ml) followed by two additional PBS-K^+^ washes and mounted with a drop of Mowiol for confocal imaging. In some experiments, T cells were stained against Kv1.3 (anti-Kv1.3 1/200, Neuromab) in combination with wheat germ agglutinin (WGA, plasma membrane marker) and calnexin (ER marker; anti-calnexin 1/100; BD Transduction Laboratories) as described^37^.

CY15 dendritic cells were cultured on 20 × 20 mm poly-d-lysine-coated glass coverslips for up to 24 h. Dendritic cells were fixed in methanol (-20 °C) for 15 min at RT, washed twice with PBS-K^+^ for 5 min to rehydrate cells and blocked and permeabilized for 1 h with blocking solution. Cells were subsequently incubated with polyclonal anti-KCNE4 (1/100, Proteintech) for 2 h, and excess antibody was removed with wash buffer. Coverslips were exposed to secondary Cy3 antibody (1:200, Molecular probes) for 1 h. After washing to remove unbound secondary antibody, CY15 cells were blocked again for 1 h using the blocking solution. Cells were incubated with anti-Kv1.3 (1/50, NeuroMab) at 4 °C overnight. Dendritic cells were washed and exposed to Cy5 secondary antibody (1/200, Molecular probes) for 1 h. After removing unbound secondary antibody, coverslips were mounted with Mowiol and examined with a confocal Leica TCS SL laser scanning fluorescence microscope (Leica Microsystems). All experiments were examined with a 63X oil-immersion objective lens (NA 1.32). Adequate filters were used, and offline image processing was performed using ImageJ (v1.53.e) software^17^. Mander's overlap coefficient (MOC) was used to evaluate signal colocalization. MOC calculates the percentage of total signal from one channel overlapping signal from the other^38^. For LPS-dependent Kv1.3 membrane surface targeting, CY15 cells were incubated for 24 h with 100 ng/ml LPS. Next, the cells were kept on ice for 2 min and processed for WGA (cell surface marker) Alexa Fluor™ 555 conjugate (Thermo Fisher Scientific) staining prior to anti-Kv1.3 labeling as previously described^16^.

### Induced patching immunocytochemistry

Induced patching immunocytochemistry (IPI) increased the staining signal. Briefly, cells were washed with PBS-K^+^ and incubated with anti-Kv1.3 extracellular antibody (1/150) in DMEM supplemented with 30 mM HEPES for 1 h at 37 °C. Next, the cells were washed twice with PBS-K^+^ and incubated with the Cya5 antibody (1/150) in DMEM supplemented with 30 mM HEPES for 1 h at 4 °C. Cells were washed three times and fixed with 4% PFA for 10 min at RT. Finally, the cells were washed three times with PBS-K^+^ and mounted as described above. When extra labeling was needed, a second round of labeling with primary and secondary antibodies was performed after the fixation protocol, following the indications described above.

### FACS-based FRET

FACS (fluorescence-activated cell sorting)-based FRET (Förster resonance energy transfer) was performed as previously described with some modifications^39^. Measurements were performed using a FACSAria FUSION (BD Bioscience) equipped with 405-nm, 488-nm and 633-nm lasers. Briefly, to measure CFP and FRET, cells were excited with a 405 nm laser, and fluorescence was collected in the CFP channel with a standard 450/50 filter. The FRET signal was measured with a 525/50 filter. To measure YFP, cells were excited with a 488-nm laser, while emission was collected with a 530/30 filter. We gated living cells according to forward and sideward scatter (FSC/SSC) and adjusted photomultiplier tube (PMT) voltages and compensation for CFP and YFP to specifically assess FRET in double-positive cells. Subsequently, we plotted FRET vs CFP and introduced a triangular gate to determine the number of FRET-positive cells. This triangle was adjusted to cells that were exclusively cotransfected with CFP and YFP and thus were FRET-negative cells. This gating strategy directly visualizes the sensitized acceptor emission arising from excitation of the CFP donor at 405 nm.

### Electrophysiology

Whole-cell currents were recorded at RT using the patch-clamp technique in the whole-cell configuration with a HEKA EPC10 amplifier (HEKA Elektronik). PatchMaster software (v2×90.3, HEKA. https://www.heka.com) was used for data acquisition. A stimulation frequency of 50 kHz and a filter at 10 kHz were used. The capacitance and series resistance compensation were optimized. Micropipettes were made from borosilicate glass capillaries (Harvard Apparatus) using a P-97 puller (Sutter Instrument) and fire polished. Pipettes had a resistance of 2–4 MΩ when filled with a solution containing the following (in mM): 84 K^+^-aspartate, 36 KCl, 10 KH_2_PO_4_, 5 HEPES, 5 EGTA, and 3 MgCl_2_ (pH 7.25 and 275 mOsm/l). The extracellular solution contained the following (in mM): 136 NaCl, 4 KCl, 1.8 CaCl_2_, 1 MgCl_2_, 10 HEPES and 25 D-glucose (pH 7.4 and 245 mOsm/l). Cells were clamped at a holding potential of -60 mV. To evoke voltage-gated currents, cells were stimulated with 250 ms square pulses ranging from -80 to +80 mV in 10 mV steps^19^. In some experiments, 200 ms duration voltage-ramp protocols ranging from -100 to +100 mV were evoked. The peak amplitude (pA) was normalized using the capacitance values (pF). Data analysis was performed using FitMaster (HEKA) and SigmaPlot 10.0 (Systat Software, https://systatsoftware.com).

According to the solutions used, the calculated equilibrium potential for potassium was -90 mV (E_K_) using the Nernst equation. The normalized G/Gmax vs. the voltage curve was fitted using Boltzmann’s equation: G/Gmax = 1/(1 + exp(V_1/2_ − V/k)), where V_1/2_ is the voltage at which the current is half-activated, and k is the slope factor of the activation curve.

### Immunological synapse formation

To characterize IS formation, human B cell lymphoma Raji cells were used as APCs. Raji cells were pulsed with 10 µg/ml *Staphylococcus* enterotoxin E (SEE, Toxin Technologies) for 30 min. Cell conjugates were formed by mixing Jurkat with Raji cells at a 1:1 ratio and spun at 200×*g* for 1 min at 37 °C. The mixtures were plated on poly-l-lysine-coated coverslips, incubated for 15 min at 37 °C in a humidified atmosphere of 5% CO_2_ and 95% air and placed on ice for labeling. The cells were washed once with TBS (Tris-buffered saline; 25 mM Tris–HCl, 150 mM NaCl, pH 7.5) and fixed with 2% PFA in TBS for 10 min. Between steps, the cells were rinsed three times with TBS. Cells were labeled with anti-Kv1.3-FITC (Alomone, 1/50), anti-CD3 Alexa 647-conjugated antibody and anti-CD19 Alexa 488-conjugated antibody (BioLegend, 1/80). To analyze Kv1.3 IS accumulation in the presence of KCNE4, human Jurkat T cells were treated with a retroviral vector system containing mGFP-Kv1.3 or KCNE4-mCherry. Conjugates were incubated with anti-CD3 antibody (Invitrogen) followed by secondary antibody (Alexa Fluor 647, GAMIG; Invitrogen). Antibodies were diluted in TBS containing 1% BSA and incubated with the cells for 30 min. Coverslips were rinsed and mounted in Mowiol.

KCNE4 expression levels were estimated from the total mCherry cellular intensity. To quantify the accumulation of Kv1.3 channels in the immunological synapse (IS) of T cells (evaluated by CD3 polarization in the contact area of T cells and B cells), we used the following expression (accumulation ratio, AR):

$${\text{AR }}= \frac{{\frac{{\left( {\text{IIS - IBG}} \right) \cdot {\text{AREAIS}}}}{{\left( {\text{Ioutside - IBG}} \right) \cdot {\text{AREAoutside - }}\left( {\text{Iinside - IBG}} \right) \cdot {\text{AREAinside}}}}}}{{\frac{{{\text{AREAIS}}}}{{\text{AREAoutside - AREAinside}}}}}$$

where I_IS_, I_outside_, I_inside_, and I_BG_ are the mean fluorescent intensity of mGFP (Kv1.3) in the IS, outside the cell (including the membrane and intracellular region), inside the cell (only intracellular region), and background intensity detected at a cell-free area of the image, respectively. AREA_i_ denotes the area of the described sections. The regions of interest were always selected according to extracellular CD3 labeling to neglect intracellular mGFP-Kv1.3. For confocal imaging analysis, ImageJ software (v1.53.e) was used. A cell was considered Kv1.3 polarized when the value of AR was greater than 1.5.

### IL-2 production

To analyze IL-2 production in Jurkat T lymphocytes, 2–6 × 10^4^ cells/ml were seeded in 96-well plates with selected treatments 24 h after FACS sorting. Supernatants were collected at 24 and 48 h after T cell activation. IL-2 secretion was measured with an ELISA kit (eBioscience) following the manufacturer’s instructions. Supernatants were centrifuged at 1200×*g* for 10 min, and the supernatant was used to quantify the IL-2 concentration. Reactions were performed in 96-well plates coated with the capture antibody. After blocking, standards and samples were incubated with detection antibody followed by HRP–streptavidin. 1 M H_3_PO_4_ was used as stop solution. Plates were read at 450 nm^36^. In some experiments, 10 nM MgTx was further added during the PMA/PHA incubation.

### Cell size, viability and proliferation assays

Jurkat lymphocytes and CY15 dendritic cells (2 × 10^5^ cell/ml) were cultured in 100 µl of complete RPMI culture media 24 h after FACS sorting in 96-well plates. A Countess^TM^ automated cell counter (Invitrogen) was used for cell size and viability by means of trypan blue exclusion. The AlamarBlue dye (Life Technologies) was used to evaluate proliferation following the manufacturer's instructions. In some experiments, increasing concentrations of puromycin were used to monitor the proliferation of LvKCNE4 and LvScramble CY15 cells. Values of control cells, cultured in the presence of FBS during 24 h after seeding, were considered to represent 100% proliferation.

### Cell cycle and apoptosis assays

Cell cycle analysis was performed with Hoechst 33342, and flow cytometric measurements were performed using a Gallios flow cytometry instrument (Beckman Coulter, Inc.). Twenty-four hours after transfection, Jurkat T cells were collected, washed in 5 ml PBS and spun for 5 min at 200×*g*. Next, cells were resuspended in 0.5 ml PBS and incubated for 30 min with a 5 µg/ml final concentration of Hoechst 33342, which stains DNA in living cells. Samples were kept for 30 min at RT.

To analyze apoptosis, cells were labeled with fluorochromes Annexin V and 4′,6-diamidino-2-phenylindole (DAPI). Thus, viable (Annexin V^−^/DAPI^−^), early apoptotic (Annexin V^+^/DAPI^−^), late apoptotic (Annexin V^+^/DAPI^+^) and necrotic (Annexin V^-^/DAPI^+^) cells can be distinguished. Briefly, 24 h after transfection, cells were centrifuged (200×*g*, 5 min), washed with 5 ml of PBS and collected. Cells (1 × 10^6^) were resuspended in 100 µl BB (Binding Buffer; 10 mM HEPES, 140 mM NaCl, 2.5 mM CaCl_2_, pH 7.4) and incubated with 5 µl of Annexin V APC (Immunotools) for 15 min at RT in darkness. Next, 400 µl of BB and 3 µl of DAPI (1 mg/ml) were added to each sample. The acquisition of events was performed in a Gallios flow cytometry instrument (Beckman Coulter, Inc.) using specific software.

### Lipid raft isolation

Low-density, Triton-insoluble complexes were isolated as previously described^10,20^ from Jurkat T lymphocytes and CY15 dendritic cells. Cells were homogenized in 1 ml of 1% Triton X-100, and sucrose was added to a final concentration of 40%. A 5–30% linear sucrose gradient was layered on top and further centrifuged (260,000×*g*) for 20 h at 4 °C in a Beckman SW41 rotor. Gradient fractions (1 ml) were collected from the top (1 to 12) and analyzed by western blotting. Flotilin identified buoyant lipid rafts, whereas clathrin suggested no floating sucrose fractions.

### Statistics

Data are presented as the mean ± SEM. Statistical analysis was performed by Student’s *t*-test and one-way ANOVA with Tukey’s post hoc test. A value of p < 0.05 was considered significant.

## Supplementary Information

Supplementary Information.
