# Supplementary material for: KCNE4-dependent functional consequences of Kv1.3-related leukocyte physiology
Source: Sci Rep. 2021 Jul 16;11:14632. doi: 10.1038/s41598-021-94015-9 (PMC8285421; doi:10.1038/s41598-021-94015-9)
Supplement: Supplementary file 1 — Supplementary Information. [file 41598_2021_94015_MOESM1_ESM.pdf]

Supplementary Information

KCNE4-dependent functional consequences of Kv1.3-related leukocyte physiology

Albert Vallejo-Gracia[1,2], Daniel Sastre[1], Magalí Colomer-Molera[1], Laura Solé[1,3], María Navarro-Pérez[1], Jesusa Capera[1], Sara R. Roig[1], Oriol Pedrós-Gámez[1], Irene Estadella[1], Orsolya Szilágyi[4], Gyorgy Panyi[4], Péter Hajdú[4], Antonio Felipe[1]

[1]Molecular Physiology Laboratory, Dpt. de Bioquímica i Biomedicina Molecular, Institut de Biomedicina (IBUB), Universitat de Barcelona, Avda. Diagonal 643, 08028 Barcelona, Spain. [2]Virology and Immunology, Gladstone Institutes, University of California San Francisco, San Francisco, CA 94158; [3]Department of Biomedical Sciences, Colorado State University, Fort Collins, Colorado 80523. [4]Department of Biophysics and Cell Biology, Faculty of Medicine, University of Debrecen, 400, 1 Egyetem sq., Debrecen, 4032, Hungary.

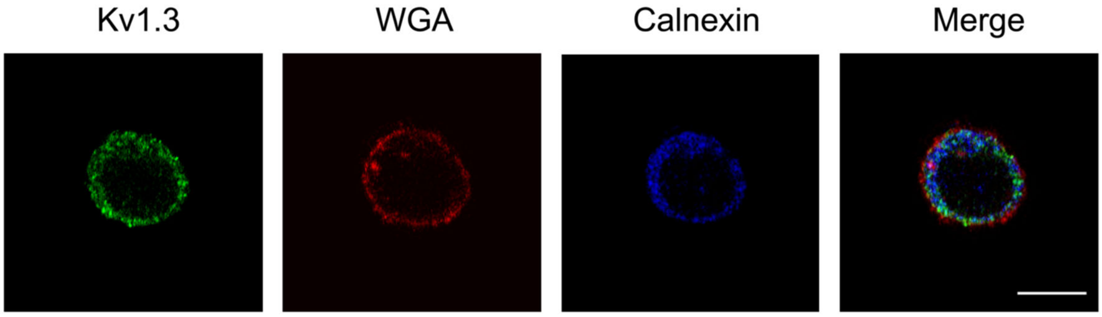

Supplementary Figure 1. Cellular localization of Kv1.3 in Jurkat T lymphocytes. T-cells were stained against Kv1.3 (green) in combination with WGA (membrane surface in red) and calnexin (ER in blue). Bar represent 10 μm.

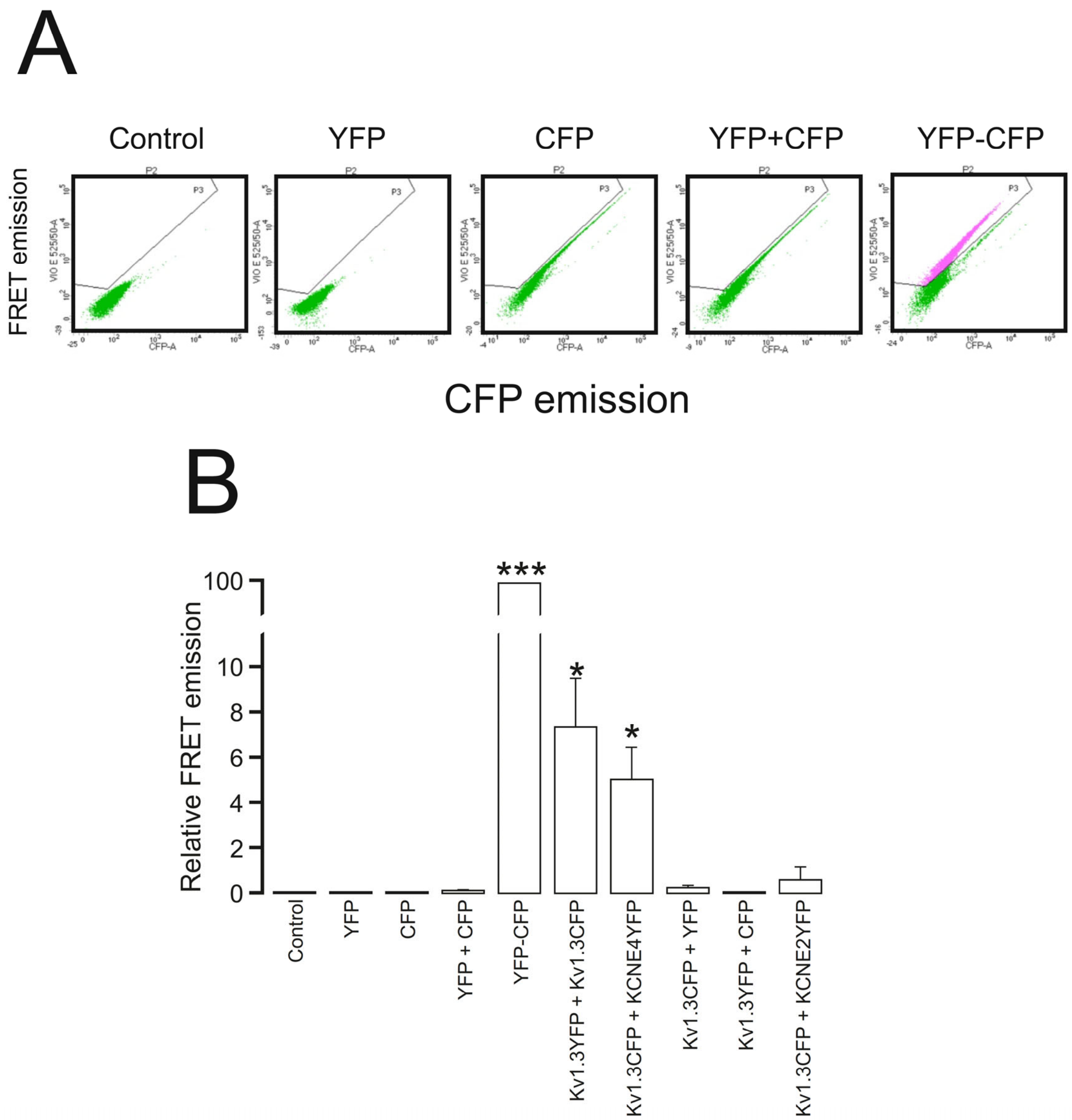

Supplementary Figure 2. Jurkat T lymphocytes were transiently transfected with different cDNAs, and FACS (fluorescence-activated cell sorting)-based FRET (Förster resonance energy transfer) was performed. Cells were electroporated. Positive transfected cells were selected 24 h later, and FRET intensity was monitored. (A) Setup of FRET measurements by flow cytometry. FRET emission (VIO E 523/50-A) shows the YFP fluorescence intensity after CFP excitation. Representative experiments of YFP-, CFP-, YFP+CFP (negative control)- and YFP-CFP (positive control)-transfected cells are shown. Magenta in YFP-CFP shows positive FRET emission. (B) Analysis of protein interaction by FRET. Relative FRET emission to YFP-CFP condition. While Kv1.3YFP + Kv1.3CFP represents a positive association, Kv1.3CFP + KCNE2YFP highlights a negative interaction. Mean ± SE. (*$p < 0.05$; ***$p < 0.001$; vs YFP + CFP; $n = 3$-7; Student's *t*-test).

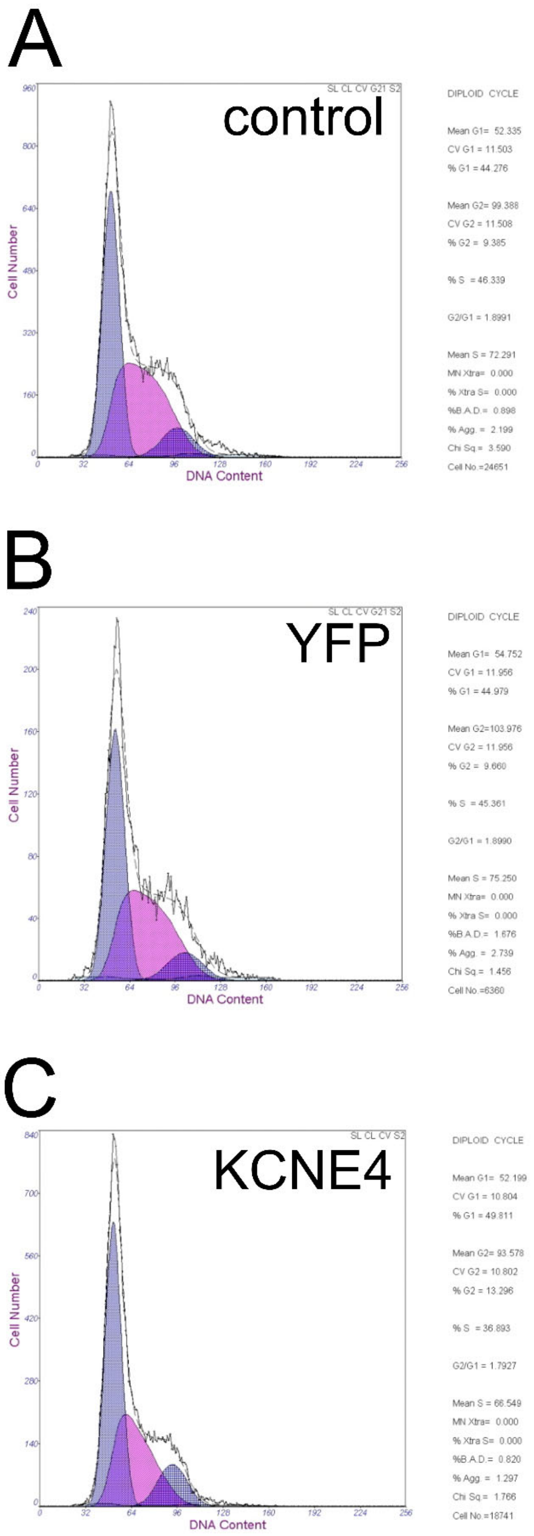

Supplementary Figure 3. Overexpression of KCNE4 impairs the cell cycle progression in Jurkat T lymphocytes. Jurkat T cells were electroporated without (control) or with KCNE4CFP or YFP, and positively transfected cells were selected for specific assays. Serum starved resting cells were incubated for 24h after serum re-addition. Cell cycle analysis of Jurkat T lymphocytes was performed with Propidium Iodide. Representative raw data histograms of control (A), YFP (B) and KCNE4CFP (C) cells. Cells appear with two blue peaks corresponding to the G_0/G_1 (left) and G_2 (right) phases, respectively. The cell population in purple corresponds to S phase.

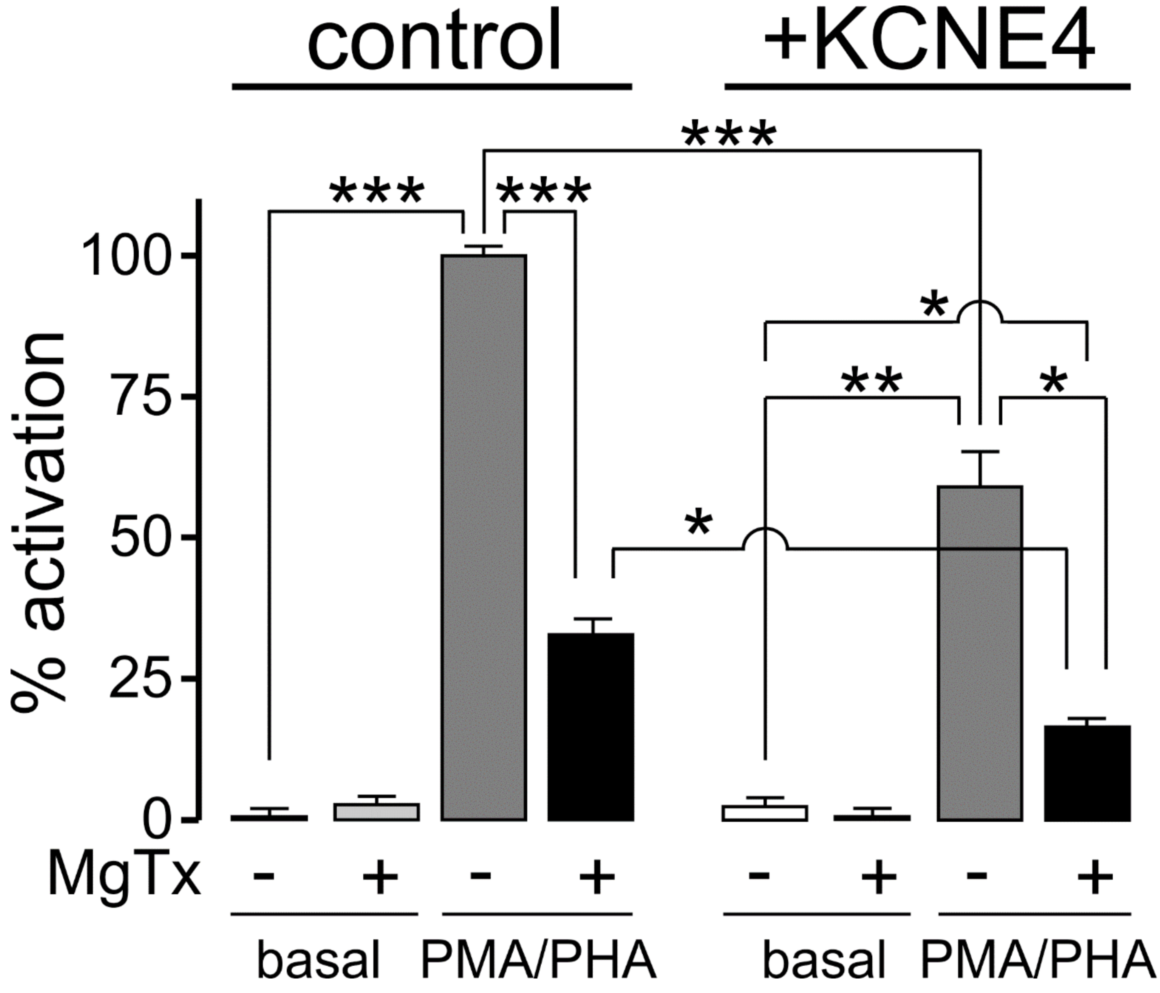

Supplementary Figure 4. Margatoxin triggers synergistic effects on the KCNE4-dependent modulation of Kv1.3-related activation of Jurkat T lymphocytes. Jurkat T cells were electroporated with KCNE4CFP, and positively transfected cells were selected for IL-2 production assay. Margatoxin (MgTx) as well as KCNE4CFP overexpression impaired IL-2 production in activated Jurkat T lymphocytes. Cells were cultured in the presence or absence of PMA (80 nM) and PHA (5 mg/ml) with (+) or without (-) MgTx (10 nM) for 48 h. IL-2 production was measured in all groups and the percentage of the cytokine production was relativized to the maximum production in the PMA/PHA group. Values are mean ± SE of n=4-6 independent experiments each done in duplicate. * $p < 0.05$; ** $p < 0.01$; *** $p < 0.001$ (one-way ANOVA and Tukey's post hoc test). White bars, basal (no additions) without (-) MgTx; light gray, basal in the presence (+) of MgTx; dark gray, PMA/PHA incubation in the absence (-) of MgTx; black, PMA/PHA incubation in the presence (+) of MgTx. Control, Jurkat nontransfected cells; +KCNE4, KCNE4CFP-positive cells. Note that KCNE4 (+KCNE4) hampered the IL-2 production but the further addition of MgTx synergistically decreased the PMA/PHA-induced IL-2 production.

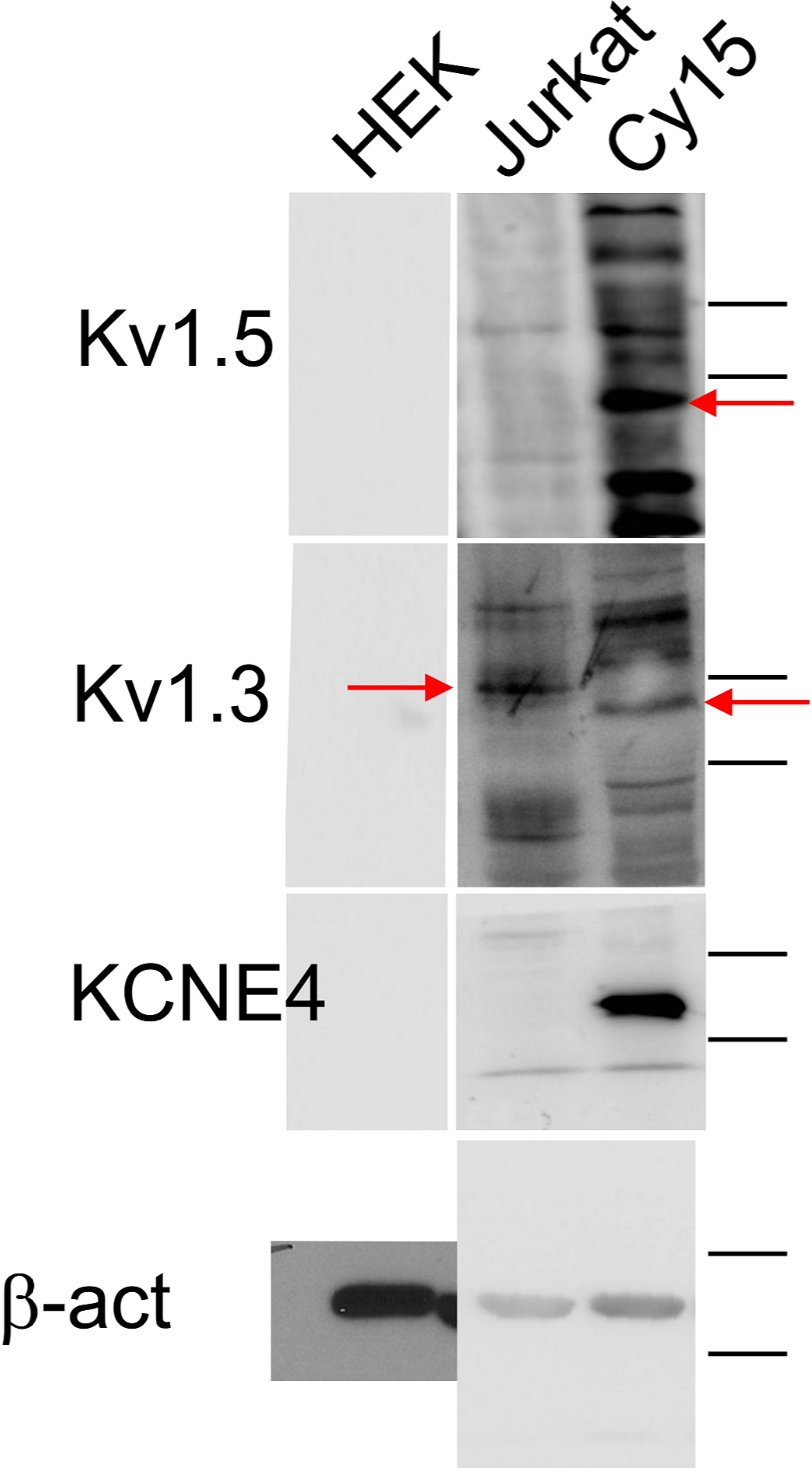

Raw data Figure 1A

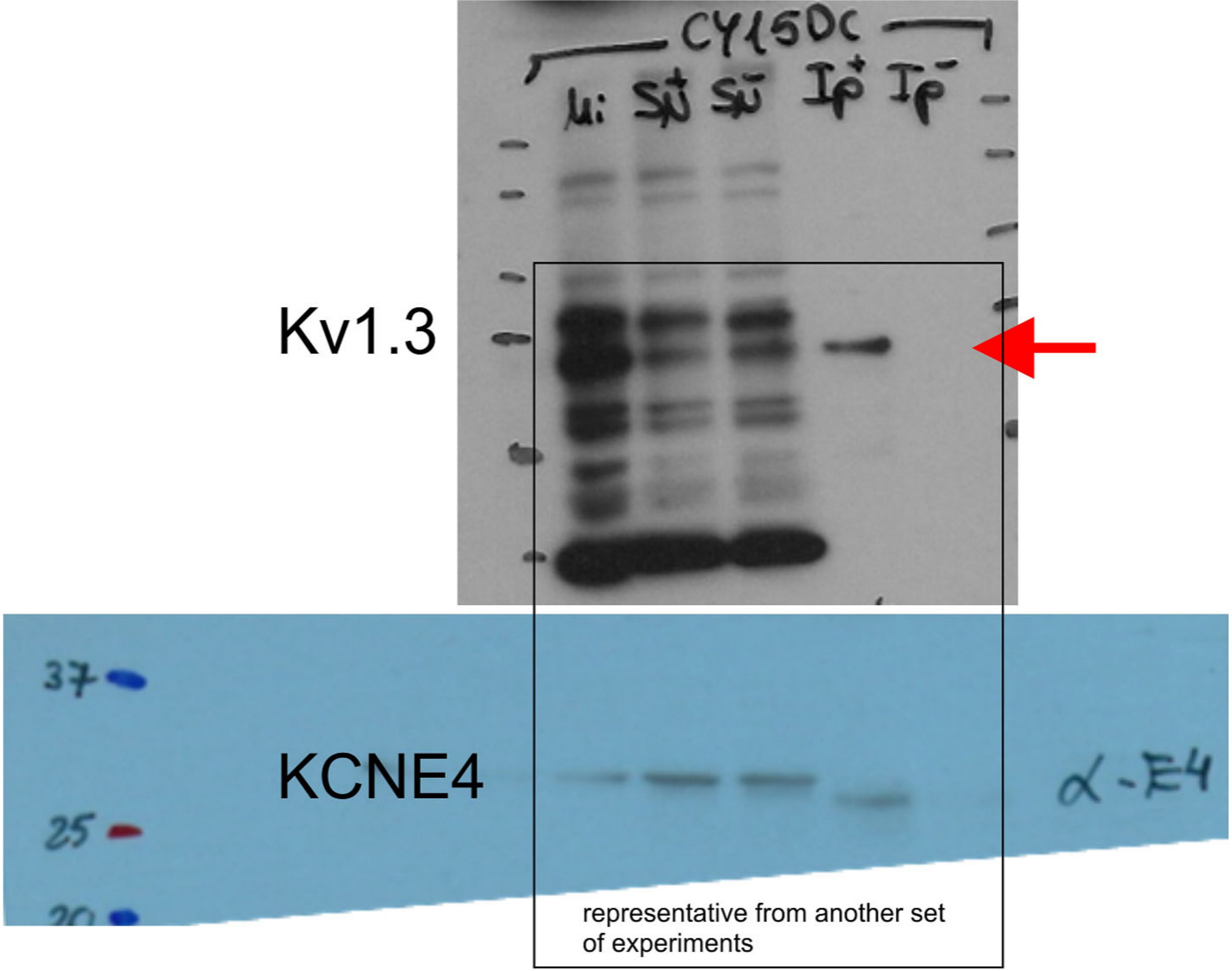

Raw data Figure 1E

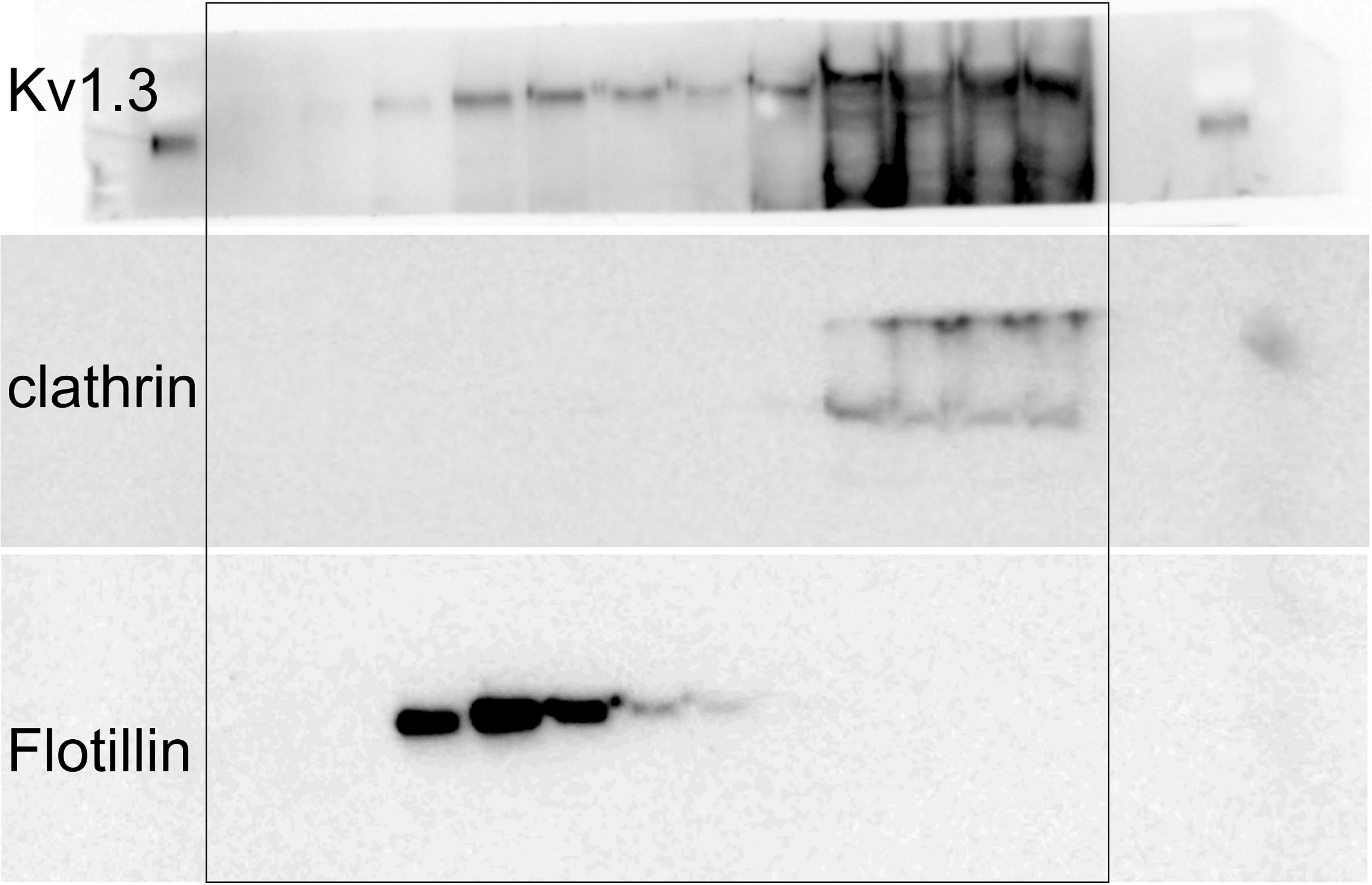

Raw data Figure 1F

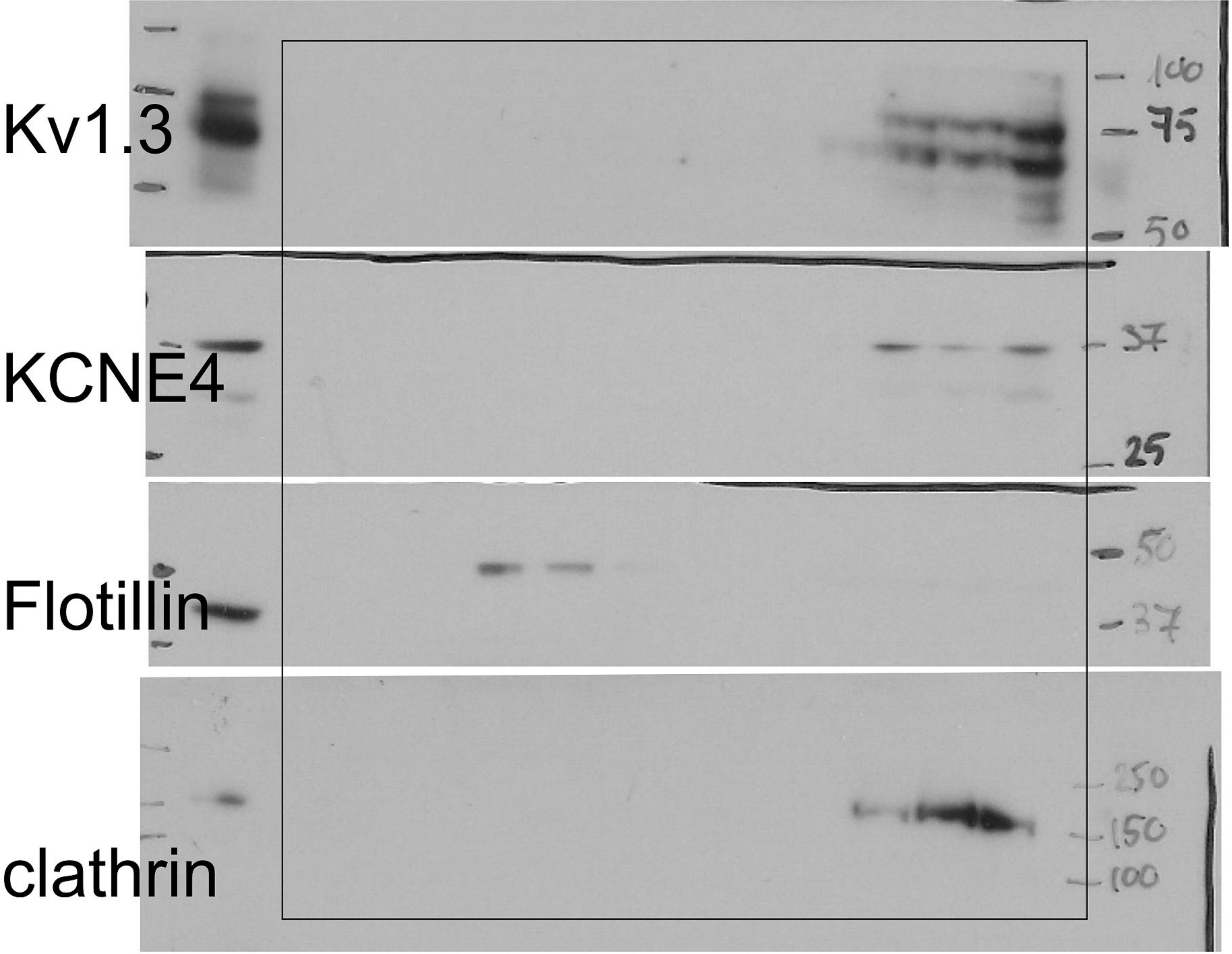

Raw data Figure 1G

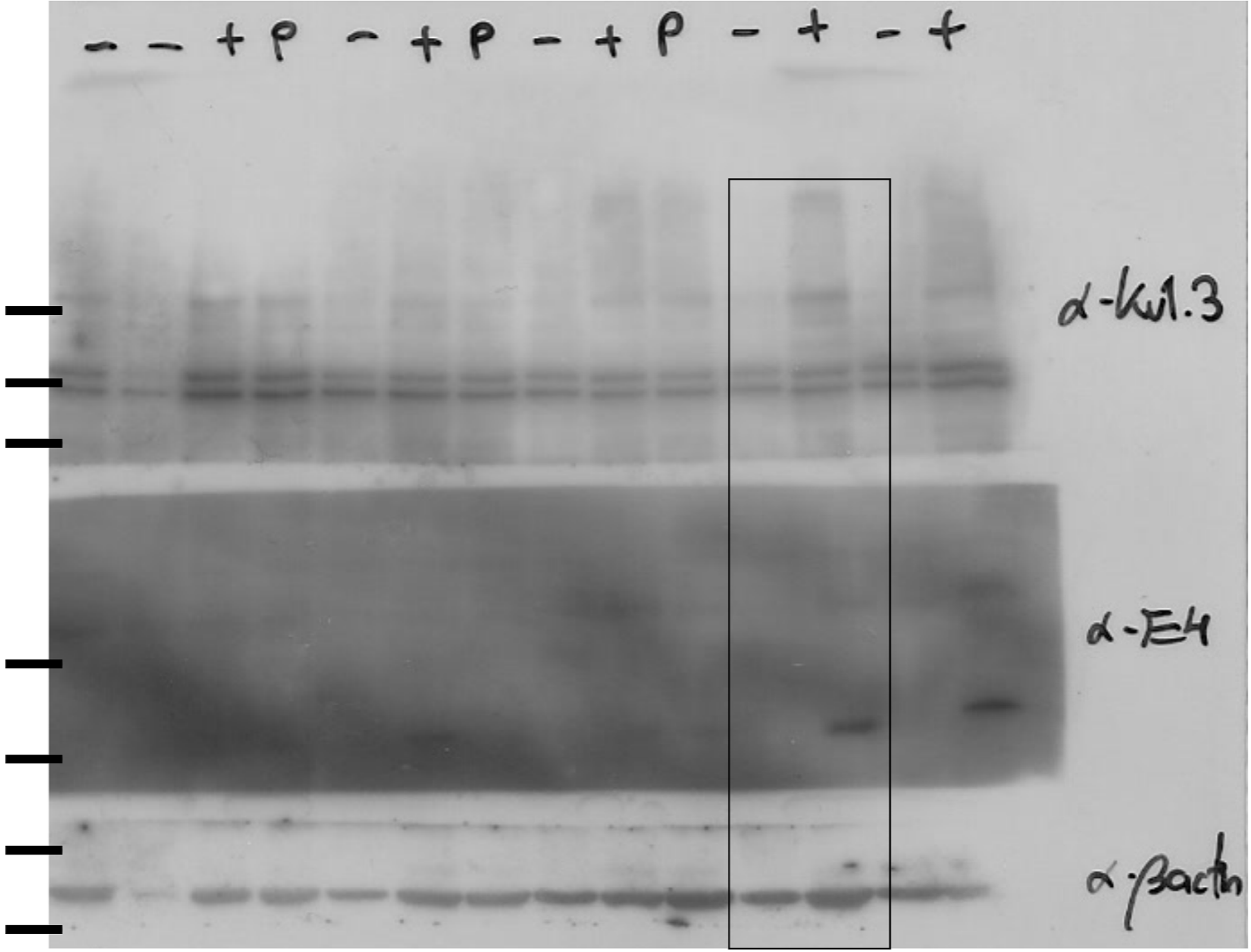

Raw data Figure 3 A

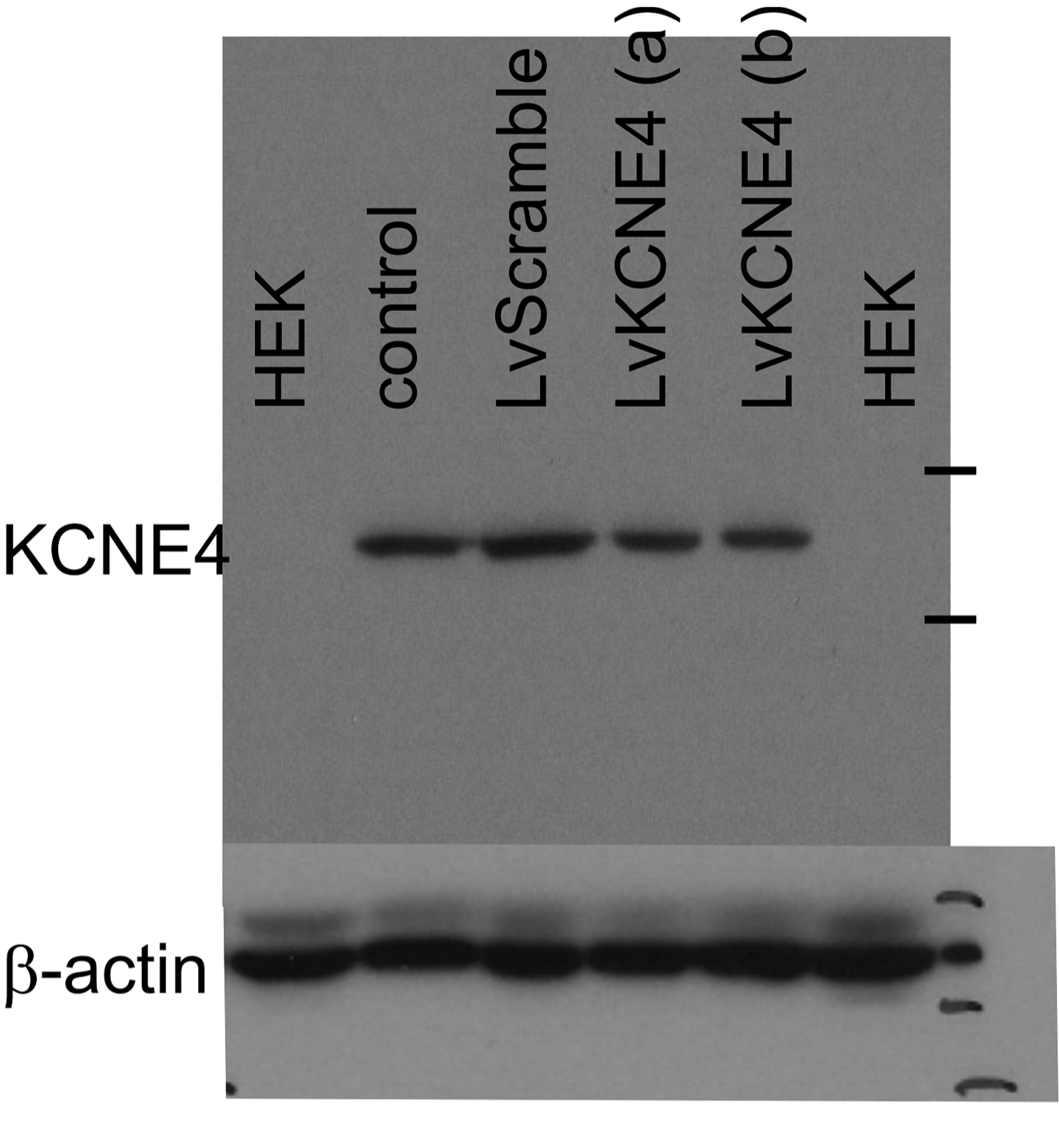

Raw data Figure 5 A

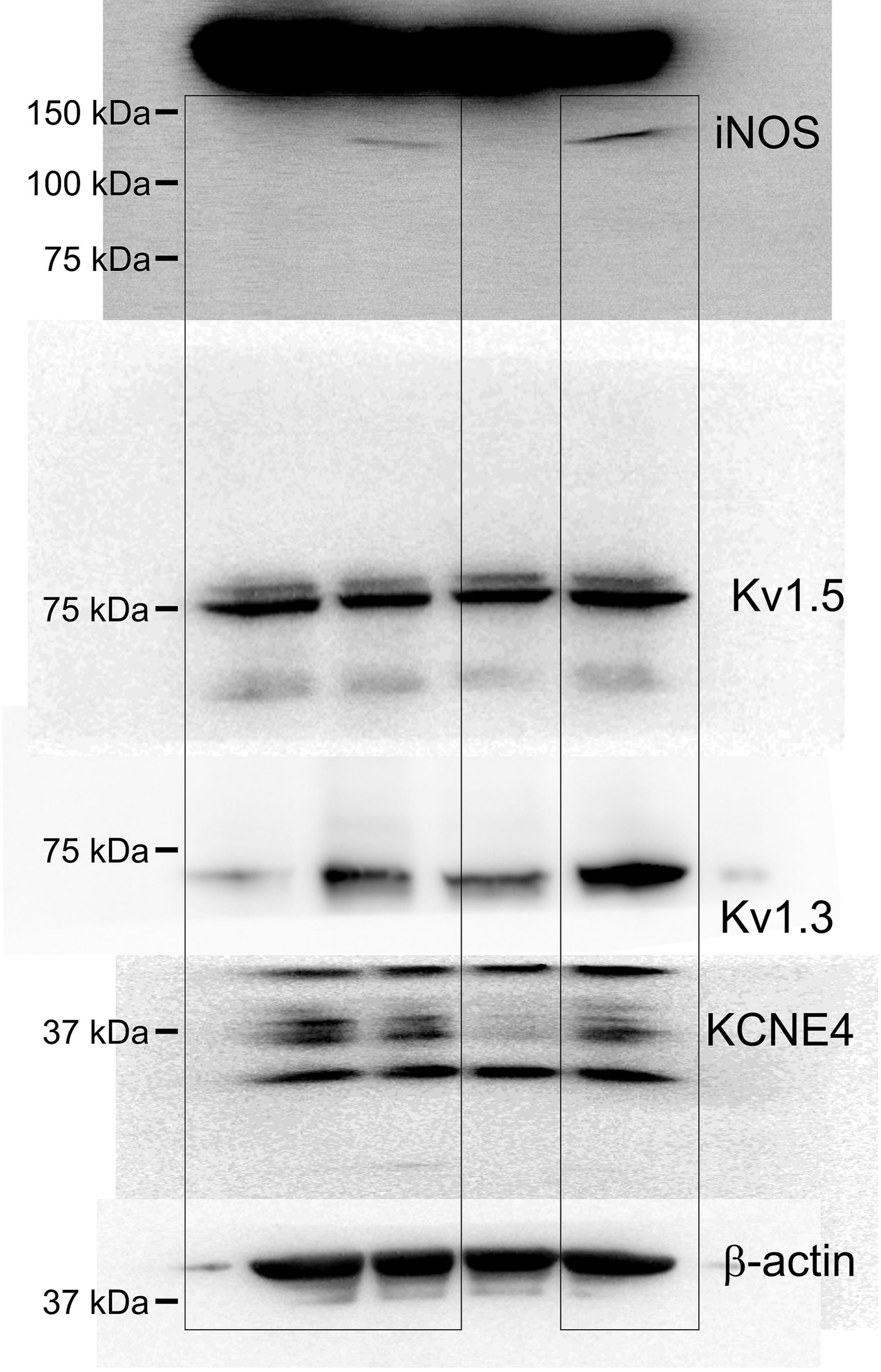

Raw data Figure 6 D

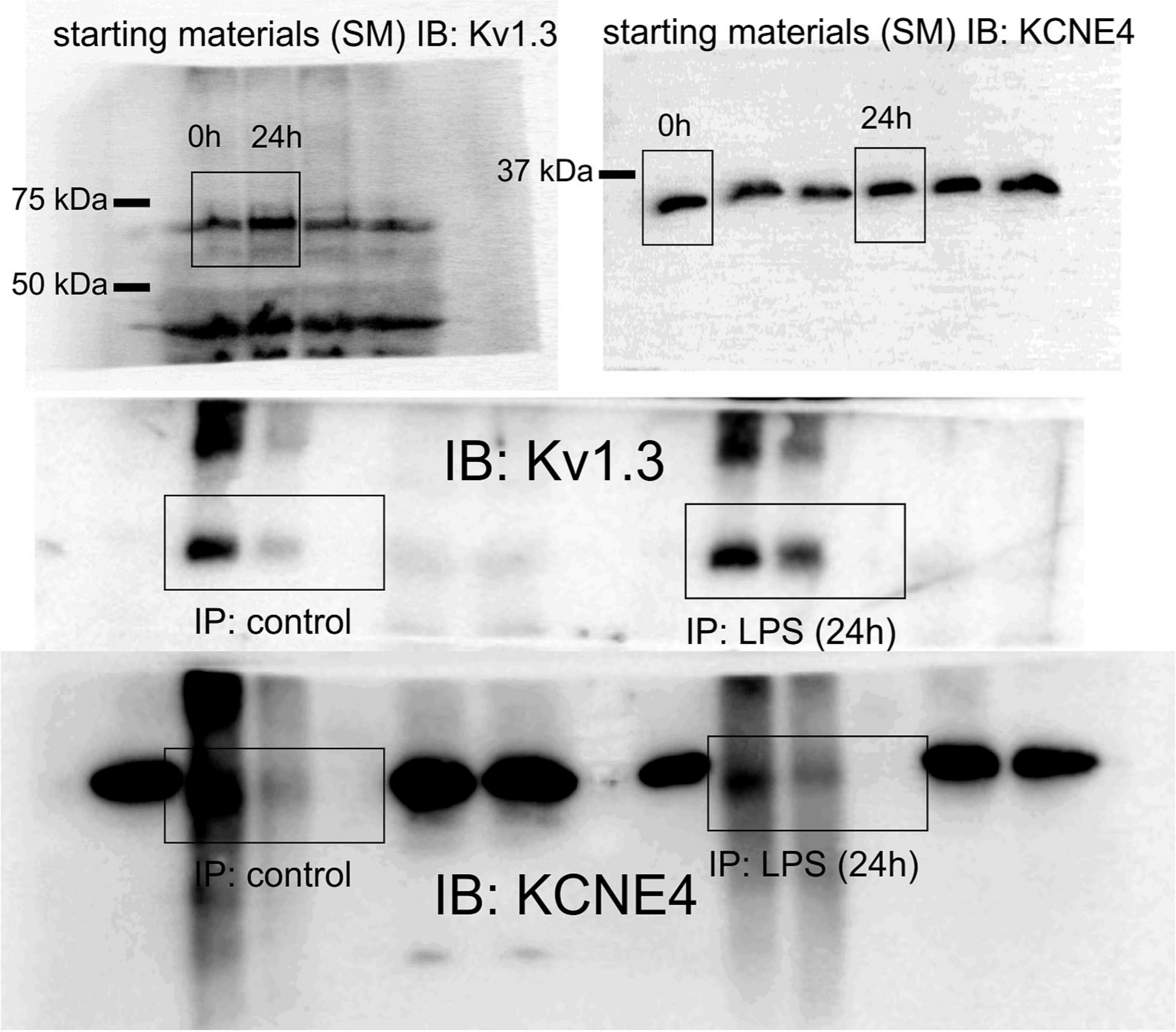

Raw data Figure 6 F
